# Changes in Hop (*Humulus lupulus* L.) Oil Content and Composition during Long-Term Storage under Different Conditions

**DOI:** 10.3390/foods11193089

**Published:** 2022-10-05

**Authors:** Ksenija Rutnik, Miha Ocvirk, Iztok Jože Košir

**Affiliations:** 1 Department for Agrochemistry and Brewing, Slovenian Institute of Hop Research and Brewing, 3310 Žalec, Slovenia; 2 Biotechnical Faculty, University of Ljubljana, 1000 Ljubljana, Slovenia

**Keywords:** hops, storage stability, hop essential oil, hop oil composition

## Abstract

Hop essential oil, in addition to alpha-acids, is one of the most valuable parameters for brewers, since it is responsible for beer aroma. The hop oil content and hop oil chemical composition deliver various aromas to beer. During storage, the hop chemical composition undergoes many physical and chemical changes that impact its quality. The main purpose of our study was to evaluate the changes occurring in hop oil content and its chemical composition during two years of storage under four different conditions (anaerobic, aerobic, cold room (4 °C), and room temperature) in the form of cones and pellets, supplied by local suppliers in Slovenia. Hop oil content and composition were determined by steam distillation and GC-FID. The greatest decrease in hop essential oil content occurred when the hops were stored under aerobic conditions and at room temperature. Monitoring of eight hop oil compounds under various conditions revealed different behaviors of the changes. The best storage conditions were anaerobic at low temperatures. Oxygen resistance was lower for pellets than for hop cones, whereas high temperature was more devastating for hop cones. In addition to the storage conditions, the hop variety and form were important factors regulating the extent of changes in hop oil content and chemical composition.

## 1. Introduction

Beer aroma plays a key role in our perception of beer quality and is a product of a synergistic effect between compounds found in hops, barley malt, and yeast. Barley malt gives beer its body, while higher alcohols, esters, and vicinal diketones produced by yeast are compounds that also have a great impact on the final aroma of beer [1,2]. A third factor influencing beer aroma arises from the components found in hop essential oil [3]. Based on their concentrations and ratios, they can impart various aromas, such as citrussy, herbal, floral, fruity, or typical hoppy aromas. When hops are harvested and processed, their levels of essential oil start to decrease, and the chemical composition of the oil starts to change. These changes can be so extensive that the aroma they deliver to beer is of a different type [4]. The changes can be slowed down with proper storage; however, despite the fact that awareness of the importance of proper storage is growing, it is still not adequate. Some hop growers still do not have cold rooms, and their hops—if they are not delivered to the breweries immediately—are definitely not of the best quality. In addition, in 2019 and 2020, the supply of hops exceeded the demand by the brewing industry, resulting in a hop surplus [5]. Part of the delivered supplies was used one or even two years after harvesting, and some of it is still in storage. Despite the awareness that the quality of those supplies is not as high as that of fresh hops, little is known regarding the changes in hop oil chemical composition over two years of storage. Even less information is available regarding the influence of different storage conditions. Previous research investigating hop storage stability was mainly focused on levels of alpha-acids and hop storage index (HSI) during storage.

In 1977, Skinner et al. [6] already investigated the effect of storage temperature on the stability of baled hops. The alpha-acid content of the hops was found to decrease at a linear rate, at each storage temperature (−20 °C, −5 °C, +5 °C, +22 °C). The lower the temperature, the higher the preservation of alpha-acids. Mikyška and Krofta [7] evaluated the changes of hop resins and polyphenols during 1-year storage in hop pellets of four different varieties. Storage at low temperatures without air access turned out to have minor effects on alpha- and beta-acids. Storage at 20 °C with air access led to a large decrease in resins and polyphenols. In addition, the hop variety showed to be an important factor in storage stability. Since the amount of studies investigating hop oil content and composition during storage is few, we take a look into other plant essential oils also. Kumar et al. [8] investigated the oil content and quality of damask rose flowers under different storage conditions. Only the storage temperature was variable in storage conditions, and it significantly affected the oil content. The highest temperature (25 °C) reflected the lowest yield. However, short storage of flowers was found to increase the citronellol and nerol levels, which are preferred in the rose oil trade. Ebadi and others [9] investigated the influence of packaging methods (packaged with air, nitrogen, or under vacuum) and storage duration (0, 2, 4, 6, 8 months) on the oil content of lemon verbena. Packaging with nitrogen preserved the highest essential oil content at the end of 8-month storage, and vacuum turned out better than air presence. Summarized, low temperatures with no access to air are better for obtaining hop oil amount and hop oil composition.

However, we also found some research regarding hop oil and its composition during storage. In 1985, R.T. Foster and Nickerson [10] evaluated the chemical changes in hop oil content and composition during 6 months of storage at ambient temperature and found that 20 different varieties lost 27.6–90.0% of oil. They sorted their compounds into four groups: major hydrocarbons, oxidation products, floral compounds, and citrussy compounds. The major hydrocarbon levels declined in all varieties, while the levels of oxidation products rose. The content of citrus group oils decreased, while the floral group increased in the majority of the varieties. Tedone et al. [11] investigated the changes in hop oil content and the changes in 44 hop oil compounds within one month of storage under ambient temperature and aerobic conditions. They investigated three varieties in the form of hop cones and pellets and found that hop cones lost from 53 to 62% of the oil and pellets lost from 43 to 58% after 30 days. In the three varieties, the decrease was greater for hop cones than for hop pellets. Among the hop oil compounds, beta-myrcene, alpha-pinene, beta-pinene, and limonene concentrations decreased, whereas the levels of other compounds, such as humulene epoxide and caryophyllene oxide, increased. Canbaş et al. [12] investigated the effects of storage temperature (anaerobic conditions) on the chemical composition of hop pellets of five different varieties. Storage of the hop pellets at 3 °C and room temperature for 6 months confirmed that both the storage conditions and the hop variety play important roles in hop stability. The hops lost 4.4–62.5% of their oil when stored at 3 °C and 27.6–90.0% when stored at room temperature.

Overall, the loss of hop oil and the differences in chemical composition are correlated with the hop variety, but proper storage conditions can slow down the losses and changes. However, we have not found any study that has monitored these losses and changes for durations longer than 6 months, and no study has compared more than two types of storage modes. In times of hop surplus on the market, greater knowledge is needed regarding the changes in hop essential oil during storage, since those changes impact beer quality. Rutnik et al. [13] investigated the impact of hop freshness on dry-hopped beer quality. Beers brewed with hops with hop storage index (HSI—indicator of hop freshness) values greater than 0.4 had deviations in aroma and bitterness when compared with beers brewed with fresh hops. In addition, the loss of hop oil cannot be compensated with higher doses of hops, since the chemical composition is totally different from the original one. Our goal in the present study was to evaluate the changes in the content and chemical composition of hop essential oil during 2 years of storage under four different storage conditions.

The experiment was devised with six different varieties of hops, in the form of both hop cones and pellets, stored under anaerobic or aerobic conditions and at cold or ambient temperatures. Throughout the first year, the analyses were conducted monthly, but after one year, the period was extended to two months. The selected regime of the experiments is based on the fact that the major changes are expected in the first year and on the fact that the majority of the hop is sold and used within the first year, so more frequent monitoring is of great importance for hop growers, merchants, and brewers. At each sampling, the oil content was monitored, and the content of eight components was analyzed by gas chromatography–flame ionization detection (GC-FID) to evaluate the changes in hop oil compounds.

## 2. Materials and Methods

### 2.1. Materials

Methanol (HPLC grade) was purchased from J. T. Baker (Deventer, the Netherlands); hexane was obtained from Honeywell (Charlotte, NC, USA); alpha-pinene (98%), myrcene (99%), linalool (97%), geraniol (98%), beta-caryophyllene (99%), alpha-humulene (CRM), and caryophyllene oxide (95%) were purchased from Sigma Aldrich (St. Louis, MO, USA).

Six different hop varieties (Celeia, Aurora, Bobek, Styrian Gold, Savinjski Golding, and Styrian Wolf) in the form of cones and pellets (Table 1) were used in the experiment. All samples were manufactured in October 2019, with a shelf life of three years. Celeia, a variety bred at the Slovenian Institute of Hop Research and Brewing (SIHRB), is known for its noble and hoppy aroma. In addition to Celeia, Aurora is the most commonly grown hop variety in Slovenia. It has a high brewing value with alpha-acid levels between 7.2 and 12.6% and an intense hoppy aroma. Bobek also has an intense and pleasant hoppy aroma and contains 0.7–4.0 mL/100 g of essential oils. Styrian Gold was bred to improve the agronomics of Savinjski Golding [14]. Styrian Wolf is one of the latest varieties bred by SIHRB, with a very intense aroma, high oil content, and high alpha-acid content due to the excellent transfer of aroma into beer. It is frequently used in dry hopping [15].

A 140 g sample of each variety was weighed and packed into bags (PET/Al/LDPE, 12/8/10 μm, with matt Al side outside). For each variety, four different types of packages were prepared: two vacuum sealed and two non-vacuum sealed. One of each type was stored in a cold room (4 °C), and the others were left at ambient temperature in a warehouse where unsold hops are stored throughout the year, when the use of a cold room is not possible. A sufficient number of samples was prepared to allow monthly analysis for two years. In total, 384 samples were packed and stored under the desired conditions.

### 2.2. Sampling

All sample types (all varieties, all forms, and all storage conditions) were analyzed monthly in the first year of the experiment. In the second year, the analysis period was extended to once every two months. In every sampling round, 40 samples were analyzed. All experiments were performed in duplicate.

### 2.3. Moisture Content

The moisture content of all samples was determined strictly following the official Analytica-EBC 7.2 method for moisture content of hops and hop products [16]. In brief, samples were weighed, dried at 103 to 104 °C for 1 h, and weighed again after cooling to room temperature. Moisture was calculated as the mass difference.

### 2.4. Content of Hop Essential Oil

The content of hop essential oil was determined using a steam distillation technique following the procedure prescribed in Analytica-EBC 7.10 [17]. Briefly, 50 g of ground hops was weighed into a 2 L flask, and 1 L of distilled water was added. The flask was then attached to the distillation apparatus, and the trap was filled with water. Distillation was carried out for 3 h. The volume of oil was measured, and the oil was analyzed by GC. The oil content was reported as mL per 100 g dry matter to two decimal places.

### 2.5. Chemical Composition of Hop Essential Oil by GC-FID

Eight chosen components were identified and quantified using a capillary gas chromatograph equipped with a flame ionization detector (Agilent, Palo Alto, CA, USA), following the official method in Analytica-EBC 7.12 [18]. Essential oil isolated from hop material was dissolved in hexane (0.1 mL/2 mL) and separated into individual compounds by GC on an HP-1 column (25 m × 0.2 mm, 0.11 μm, Hewlett Packard, Palo Anto, CA, USA) using helium 5.0 as carrier gas. The temperature program was as follows: 60 °C (1 min hold), 60–190 °C (2.5 °C/min, 1 min hold), and 190–240 °C (70 °C/min, 11 min hold). The temperature of the injector was 180 °C, the injection was performed in split mode (17.7:1), the volume of the injection was 1 μL, and the temperature of the detector was 280 °C. The results were expressed as the relative% of a particular compound in the essential oil. Prior to analysis, a complete validation protocol was conducted. RSDs of individual compounds were below 20%, indicating that the method is accurate.

### 2.6. Statistical Analysis

To show statistical differences between samples, hierarchical clustering analysis (HCA) was performed with the first five principal components as input data calculated on the base of all measured parameters to minimize noise in the data. The first five components accounted for a sum of variances higher or equal to 98.75%. The cluster method was based on the group average. In addition, two-way ANOVA was performed to statistically evaluate the differences between different storage conditions and the shape of hops. Data were analyzed using the OriginPro^®^ 2020b (OriginLab Corporation, Northampton, MA, USA) software package.

## 3. Results

### 3.1. Changes in the Hop Oil Content in Different Varieties under Different Storage Conditions

Immediately after hop harvesting, the amount of hop oil starts to drop, but proper storage conditions can slow this loss. However, regardless of the storage conditions, some of the oil will still be lost, which means that the aroma delivered by two-year-old hops will not be the same as that of fresh hops. In our experiment, we tested different hop forms (cones and pellets), varieties, and storage conditions to evaluate the impact of those parameters on the loss of hop oil.

#### 3.1.1. Content of Hop Oil in Celeia

**Figure 1:**
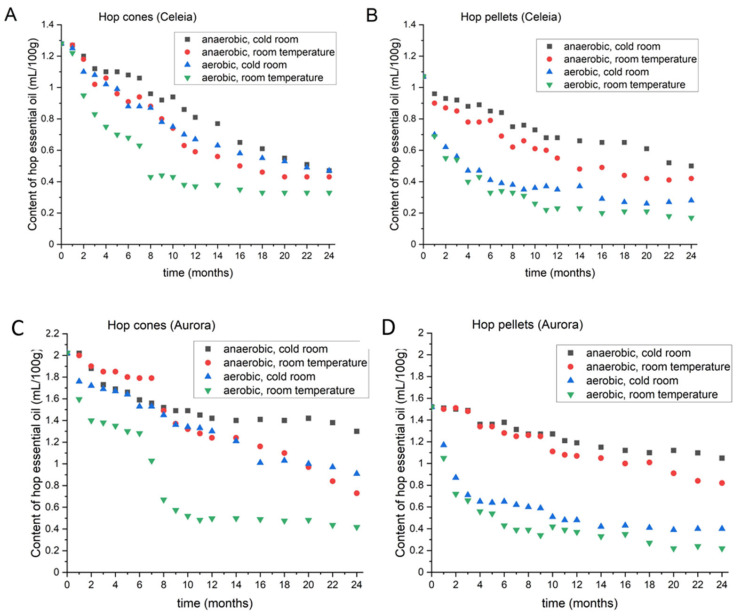
Changes in the content of hop essential oil in Celeia cones (**A**) and pellets (**B**) and Aurora cones (**C**) and pellets (**D**) during two-year period, under different storage conditions.

Figure 1 shows the hop oil content in Celeia cones (A) and pellets (B) during 2 years of storage. The initial hop oil content in Celeia cones was 1.28 mL/100 g. As shown in Figure 1A, the best storage condition for hop cones was anaerobic in the cold room at 4 °C, but 63.3% of the oil was still lost in 2 years. However, after 20 months, almost no difference was detected between storage under anaerobic vs. aerobic conditions at 4 °C. After 2 years, the difference in hops stored at room temperature, anaerobic conditions, and optimal storage was less than 5%. Storing Celeia hop cones at room temperature in the presence of oxygen resulted in a 74.2% hop oil loss. In the first eight months, Celeia lost about 50% of its oil and an additional 25% over the next 16 months. A comparison of anaerobic storage at room temperature and aerobic storage at 4 °C revealed no significant difference in the first 10 months, but storage under aerobic conditions at 4 °C was subsequently more beneficial. With pellets (Figure 1B), the results were not exactly the same, as a difference was seen between storage under anaerobic conditions at room temperature and under aerobic conditions at 4 °C even in the first month of storage. For pellets, therefore, storage under anaerobic conditions is crucial. The initial pellet hop oil content was 1.07 mL/100 g, and it decreased to 0.50 under combined 4 °C and anaerobic conditions. At room temperature, the oil content declined to 0.42 and to 0.28 compared to aerobic and 4 °C conditions, respectively. Nearly 85% of the oil was lost when pellets were left in air at room temperature. With pellets, the greatest decline was observed in the first month, regardless of the storage conditions.

#### 3.1.2. Content of Hop Oil in Aurora

Figure 1C,D shows the changes in hop oil content in Aurora cones (C) and pellets (D) during 2 years of storage. An interesting pattern was evident within the first 8 months in Aurora cones when anaerobic conditions were compared at 4 °C and room temperature. The hop oil content was initially higher when the cones were stored at room temperature, but after 8 months, the level of oil drastically dropped. Cold room temperature was subsequently better. At the end of the 2-year period, the storage conditions that were the best for the first 8 months were only better than aerobic conditions at room temperature. Storage of hop cones under aerobic conditions confirmed that the greatest decline appeared within the first month. Another huge decline appeared after 6 months when the hop cones were stored under aerobic conditions at room temperature. After 2 years, the Aurora cones lost 35.6% of their oil under anaerobic conditions at 4 °C, 55% under aerobic conditions at 4 °C, 63.9% under anaerobic conditions at room temperature, and 79.3% under aerobic conditions at room temperature. By comparison, the hop pellets showed no differences in hop oil content for 6 months, as no difference was observed under anaerobic conditions at 4 °C or at room temperature. After 6 months, storage at 4 °C was better for pellets. As with Celeia, a huge decline in oil content was observed under aerobic conditions, but the loss was not as great at 4 °C as at room temperature. The hop oil content in pellets declined from 1.52 mL/100 g to 1.05 mL/100 g under anaerobic conditions at 4 °C, to 0.82 mL/100 g under anaerobic conditions at room temperature, to 0.40 under aerobic conditions at 4 °C, and to 0.22 in pellets under aerobic conditions at room temperature.

#### 3.1.3. Content of Hop Oil in Bobek

**Figure 2:**
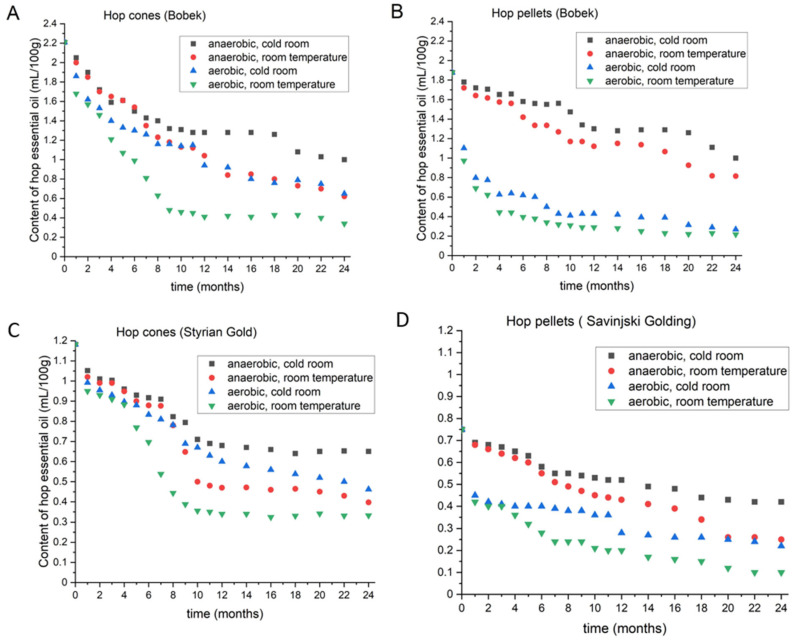
Changes in the content of hop essential oil in Bobek cones (**A**) and pellets (**B**), Styrian Gold cones (**C**) and Savinjski Golding pellets (**D**) during two-year period, under different storage conditions.

The Bobek cones used in the experiment had oil contents of 2.22 mL/100 g, and pellets had contents of 1.88 mL/100 g. Figure 2 shows the plots for Bobek hop oil content during the 2 years of storage in hop cones (A) and pellets (B). Hop cones stored under anaerobic conditions at 4 °C and at room temperature lost the same amount of hop oil (0.6 mL) during the first six months. The decline in hop oil under aerobic conditions was subsequently larger at room temperature and showed a declining trend at 4 °C. Once again, the highest amount of oil (84.6%) was lost from cones under aerobic conditions at room temperature. The oil content showed a continuous drop for 8 months and then showed only minimal changes. Under anaerobic conditions at 4 °C, Bobek cones lost 54.8% of their oil. By contrast, pellets under the same conditions lost 46.8%, indicating that pellets were more stable than cones when given proper storage conditions. Pellets under aerobic conditions lost the greatest amount of oil in the first 4 months, then the losses slowed. The final loss from Bobek pellets was 88.4%, which was slightly greater than the losses from cones under the same conditions.

#### 3.1.4. Content of Hop Oil in Styrian Gold and Savinjski Golding

We were unable to obtain cones of Savinjski Golding for our experiment; therefore, we investigated Styrian Gold under different storage conditions. Styrian Gold was bred to improve the agronomics of Savinjski Golding. Figure 2 shows the losses in hop oil content for Styrian Gold cones (C) and Savinjski Golding (D) pellets. The initial oil content in Styrian Gold cones was 1.18 mL/100 g. Under anaerobic conditions at 4 °C, the cones lost 0.53 mL of oil per 100 g during the 2 years of storage. Once again, in the initial 8 months, storage under anaerobic conditions at room temperature was better than storage under aerobic conditions at 4 °C. After eight months, the opposite effect was observed. After two years under anaerobic conditions and room temperature, the cones lost 66.4% of their oil, whereas cones under aerobic conditions at 4 °C lost 60.9% of their oil. Savinjski Golding pellets initially contained 0.75 mL/100 g of essential oil. After two years of storage under anaerobic conditions, the pellets lost 44.0% of their oil at 4 °C and 66.7% at room temperature. Interestingly, Savinjski Golding was the only variety that did not show a huge gap between oil content following storage under anaerobic conditions at room temperature and under aerobic conditions at 4 °C when the hops were in the form of pellets. However, during the 2 years of storage, pellets stored under anaerobic conditions at room temperature still performed better than those stored under aerobic conditions at 4 °C. The biggest loss of oil, at 86.7%, was again observed when pellets were stored under aerobic conditions at room temperature.

#### 3.1.5. Content of Hop Oil in Styrian Wolf

**Figure 3:**
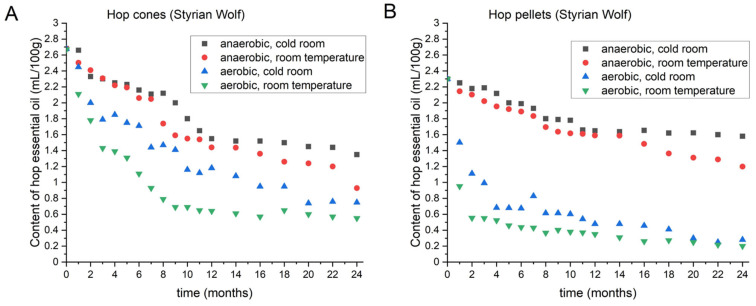
Changes in the content of hop essential oil in Styrian Wolf cones (**A**) and pellets (**B**) during two-year period, under different storage conditions.

Figure 3 shows the hop oil content during the 2 years of storage under different conditions for Styrian Wolf cones (A) and pellets (B). The initial hop oil content in Styrian Wolf was 2.68 mL/100 g for cones and 2.30 mL/100 g for pellets. Cones stored under anaerobic conditions at 4 °C lost 20.9% of their oil in the initial 8 months. Over the next 4 months, a more drastic decrease was observed, as the hop cones lost an additional 22.4% of their oil. After 2 years, the total loss was 49.6%. The dynamics of loss from the cones under anaerobic conditions at room temperature were similar to those at 4 °C until month 7. Cones stored at room temperature under aerobic conditions retained 0.55 mL/100 g of oil after 2 years. Pellets stored at 4 °C under anaerobic conditions lost 31.2% of their oil in 2 years. Once again, until a certain time (in this case, 14 months), no substantial difference was observed for pellets stored under anaerobic conditions at 4 °C or at room temperature. After 14 months, the decline was greater at room temperature than at 4 °C and accounted for 47.9% of the oil lost in the 2 years. Pellets stored under aerobic conditions and room temperature showed greater losses in the first month, and those stored at 4 °C showed greater losses in the second month. After two years, 91.3% of oil was lost from pellets stored at room temperature, and 87.8% was lost from pellets stored at 4 °C.

#### 3.1.6. Overview of the Losses of Hop Essential Oil

Table 2 shows the losses of hop oil after two years in the different hop varieties under different storage conditions. A two-way ANOVA was conducted that examined the effect of the shape and storage conditions on the content of hop essential oil after two years. There was a statistically significant interaction between the effects of shape and storage conditions on the content of oil (F(3,32) = 7.264, *p* < 0.001). There was also a statistically significant difference when the population means of different storage conditions were compared (F(3,32) = 46.021, *p* < 0.001). However, there was no significant difference between cones and pellets (F(1,32) = 0.144, *p* = 0.7). Tukey’s means comparison test was chosen for post-hoc test. As already said, there was no difference when cones and pellets were compared. There was a significant difference between all groups treated with different storage conditions. Under anaerobic conditions, regardless of the temperature, the Aurora variety performed the best, indicating that it has the best storage stability among the tested varieties. By contrast, Celeia was the least stable variety. A comparison of cones and pellets indicated that, under anaerobic conditions, oil losses were lower for pellets than for cones in all varieties. Aurora and Styrian Wolf pellets showed great storage stability, whereas Styrian Wolf cones did not.

After 2 years, all varieties lost high amounts of oil if stored at room temperature. Under aerobic conditions, the cones performed better. Under aerobic conditions at 4 °C, the smallest amounts of oil were lost from Aurora cones and from Savinjski Golding pellets. Aerobic conditions were the least appropriate for storage of Bobek and Styrian Wolf pellets, as they lost about 10% more oil than the other varieties at 4 °C. In pellets, stored under aerobic conditions at room temperature, all varieties lost more than 70% of their oil. Pellets of Styrian Wolf lost more than 90% of their oil, which means that they have practically no brewing value in terms of delivering aroma.

### 3.2. Changes in the Content of Hop Oil Compounds in Different Hop Varieties under Different Storage Conditions

Both the hop oil content and its chemical composition change during storage. The individual compounds are what impart the characteristic beer aromas, but the final aroma is a consequence of a synergistic effect between the numerous compounds found in hop oil and other beer ingredients. In our study, we monitored the behavior of alpha-pinene, myrcene, linalool, geraniol, beta-caryophyllene, alpha-humulene, beta-farnesene, and beta-caryophyllene oxide. Note that the content of individual compounds is expressed as a relative percentage, meaning that hop oil losses were not taken into account. This approach provides a more detailed look into the composition of hop oil during every month of the experiment. As in the hop oil experiment, the duration of the experiment was 2 years, the hops were in the form of cones and pellets, and the same four different storage conditions were applied as described for the hop oil content study. The amounts of data were huge; therefore, only the data for one or two varieties per compound are presented in the article; the remaining results can be found in the Supplementary Data. The comments in the article refer to all the data.

#### 3.2.1. Alpha-Pinene

**Figure 4:**
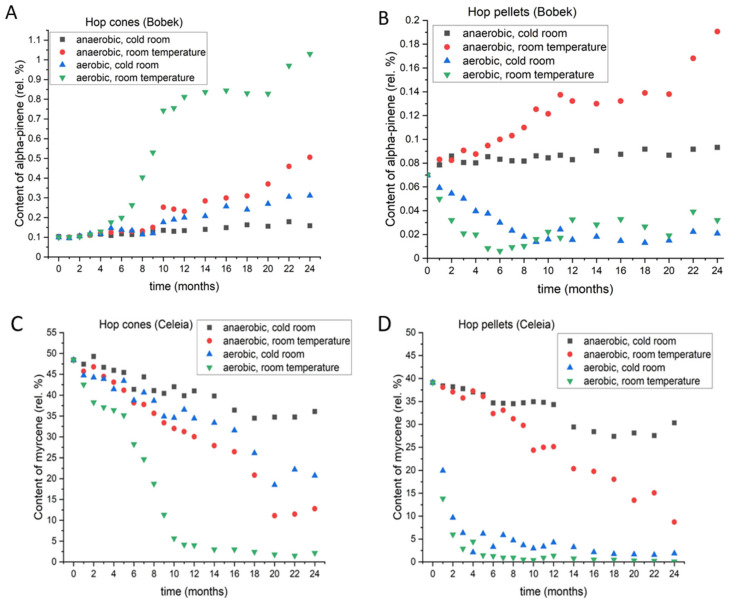
Changes in the content of alpha-pinene in Bobek cones (**A**) and pellets (**B**) and changes in the content of myrcene in Celeia cones (**C**) and pellets (**D**) during two-year period, under different storage conditions.

Alpha-pinene is a bicyclic monoterpene that delivers a woody and pine odor to beer [19]. In all four varieties used in this experiment, the levels of alpha-pinene were between 0.05% and 0.11%. Figure 4 shows the differences in alpha-pinene content during 2 years of storage under different conditions for Bobek cones (A) and pellets (B). The dynamics of changes in the other varieties followed the same patterns as seen in Bobek, and those data are presented in Supplementary Table S1. The most interesting change was observed when comparing cones and pellets. In cones, regardless of the storage conditions, the levels of alpha-pinene increased. By contrast, in pellets, the levels of alpha-pinene increased only under anaerobic conditions. When pellets were stored under aerobic conditions, the levels dropped. In cones, the levels were significantly higher at the end of the two-year period under aerobic conditions at room temperature. Under anaerobic conditions at room temperature, the rise was higher than at cold temperatures under aerobic and anaerobic conditions and lower than in cones stored at room temperature under aerobic conditions. The levels of alpha-pinene remained almost the same at 4 °C under anaerobic conditions. By contrast, pellets showed a completely different response, as the greatest increase in alpha-pinene was observed at room temperature under anaerobic conditions. The loss was similar if oxygen was present, regardless of the temperature, indicating that another very important parameter for long-term storage is the form of the hops, since the dynamics of losses and increases differ depending on whether cones or pellets are used.

#### 3.2.2. Myrcene

**Figure 5:**
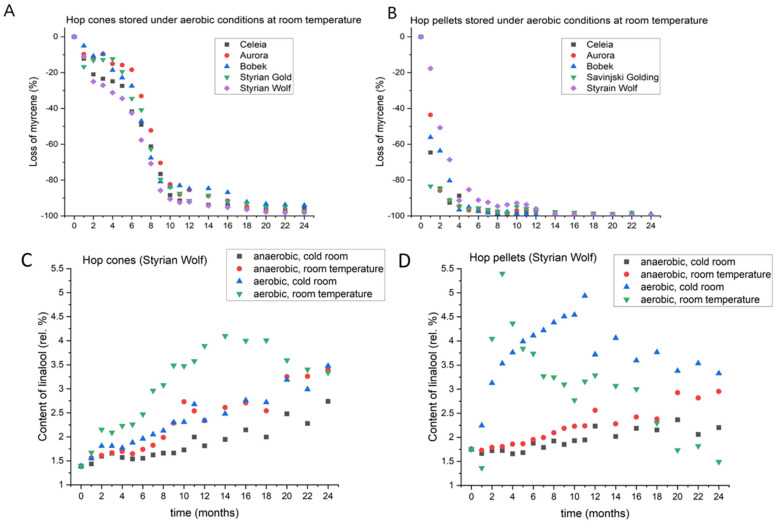
Loss of myrcene in cones (**A**) and pellets (**B**) stored under aerobic conditions at room temperature and changes in the content of linalool in Styrian Wolf cones (**C**) and pellets (**D**) during two-year period, under different storage conditions.

Myrcene, the most abundant hydrocarbon in hop essential oil, is responsible for the smell of fresh hops. It rapidly undergoes autoxidation to produce alpha-pinene, beta-pinene, geraniol, geranial, linalool, nerol, and other products [20]. Myrcene is also highly volatile, and a substantial portion is lost if storage is improper. Figure 4C,D shows the changes in myrcene content in the Celeia variety. Data for the other varieties are shown in Supplementary Table S2. Myrcene losses varied between varieties according to their storage stability, but all varieties showed the highest retention of myrcene if cones or pellets were stored under anaerobic conditions at 4 °C. Cones stored under anaerobic conditions at room temperature and under aerobic conditions at 4 °C lost slightly more myrcene in the initial eight months, but the difference subsequently increased. After 2 years, myrcene losses were lower in cones stored at 4 °C with access to oxygen than in cones stored at room temperature under anaerobic conditions. This was the case for all varieties except for Styrian Wolf, which showed a stricter requirement for storage under anaerobic conditions than for low temperatures for the retention of myrcene. Hop pellets stored under anaerobic conditions, had almost the same dynamics of loss until a certain point. That point was at 9 months for Celeia and 7 months for Savinjski Golding, but was not for 18 months for the other three varieties, indicating that pellets of Aurora, Bobek, and Wolf are more resistant to high temperatures compared to Celeia and Savinjski Golding. Another interesting point worth mentioning is the dynamics of myrcene loss in cones and pellets stored under aerobic conditions (i.e., with access to oxygen). Figure 5 shows the losses of myrcene in oil (in%) in all varieties under aerobic conditions and at room temperature for cones (A) and pellets (B). The cones were less prone to losses of myrcene in the initial months, whereas the pellets lost almost all their myrcene in the initial 4 months. A more drastic decrease in myrcene content was evident in the cones after six months; however, by then, the pellets had already lost most of their myrcene.

#### 3.2.3. Linalool

Linalool is frequently identified as a key aroma contributor, since it has good solubility and delivers floral, citrus, and fresh notes to beer [21]. Figure 5 shows the linalool content in Celeia hops (C for cones and D for pellets); data for other varieties are provided in Supplementary Table S3. The hop cones of Aurora, Bobek, and Styrian Gold did not show substantial changes in linalool content due to storage conditions for the first few months. A slightly higher content was noticed in Celeia if storage was at room temperature with access to oxygen. In Styrian Wolf, the rise in linalool was noticed earlier than in the other varieties (under aerobic and room temperature conditions). After 4–5 months, a similar change was visible in the other varieties as well. After some time, depending on the variety, the levels of linalool began to drop, and after 2 years, they were similar to those of cones stored at room temperature with no access to oxygen. The smallest increase in linalool content was observed in cones stored at 4 °C under anaerobic conditions and for pellets under anaerobic conditions at 4 °C. However, for the other storage conditions, the dynamics are not the same as observed in the cones. The greatest increase in the initial months was observed when the pellets were stored at room temperature with access to oxygen, but after some time, the levels started to drop, and, by the end of the 2-year storage period, the linalool content was the lowest among all storage conditions and for all varieties. This was exactly the opposite of the situation with cones, which showed the highest levels under those storage conditions. In Celeia and Savinjski Golding, the linalool content was highest at room temperature under anaerobic conditions in Aurora, Bobek, and Styrian Wolf, whereas the highest level was achieved under cold room and aerobic conditions. Storage of pellets under aerobic conditions at 4 °C resulted in an increase in linalool in the initial months, but the level began to decrease after approximately one year. Although high levels of linalool are desired in beer, the synergistic effect of all the other compounds that develop during storage and the fact that the aroma delivered with this combination has not yet been evaluated have to be taken into account.

#### 3.2.4. Geraniol

**Figure 6:**
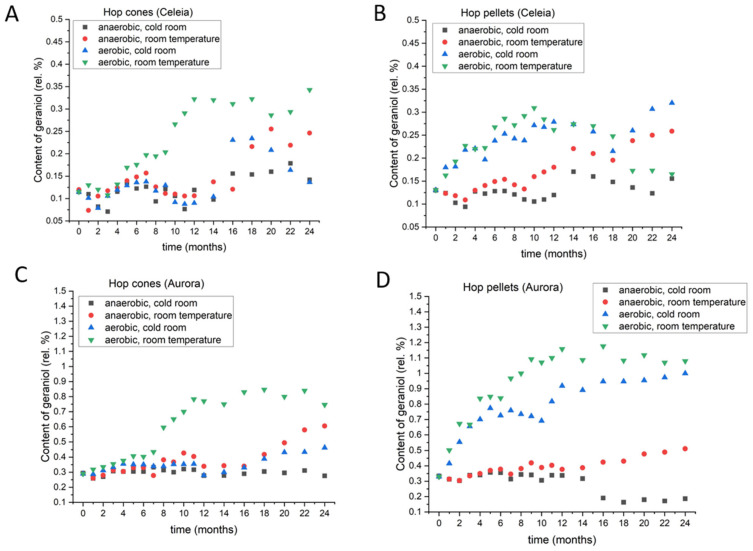
Changes in the content of geraniol in Celeia cones (**A**) and pellets (**B**) and Aurora cones (**C**) and pellets (**D**) during two-year period, under different storage conditions.

Geraniol is also frequently mentioned when it comes to beer aroma. It belongs to the family of monoterpene alcohols, and is more cultivar-specific than linalool [22], so its levels in hop oil depend on the hop variety. The changes in geraniol content for Celeia and Aurora hops and pellets are presented in Figure 6; the remaining data are provided in Supplementary Table S4. After the initial 4–6 months, a slight increase in geraniol is observed in all varieties, regardless of the storage conditions. Subsequently, the greatest increase in geraniol occurs in cones stored under aerobic conditions at room temperature. In all varieties, except for Styrian Gold, the highest levels after two years are still observed in cones stored under those conditions. Geraniol in cones stored at room temperature and aerobic conditions showed an initial increase, but the levels started to decrease after 7–10 months, followed by a rise. The length of the decrease and the start of the increase were variety dependent. For Aurora, Styrian Gold, and Styrian Wolf, the levels of geraniol slowly increased after 1 year until the second year. In Celeia and Bobek, following the initial increase, a decrease was observed. In Celeia, the increase was pronounced, and after the decrease, the levels were the same as in the cones stored under anaerobic conditions at 4 °C, where the smallest changes occurred during the 2 years of storage.

In hop pellets, the hop variety played an even bigger role than in hop cones. In Celeia, Bobek, and Styrian Wolf, the highest amount of geraniol, after 2 years, was found in pellets stored under aerobic conditions at 4 °C, whereas the highest levels were found in Aurora pellets stored under aerobic conditions and room temperature and in Savinjski Golding stored at room temperature with no access to oxygen. In Celeia and Savinjski Golding, geraniol showed a strong increase when stored under anaerobic conditions at room temperature, while the levels did not change much in the other varieties. As in the hop cones, the smallest difference from the initial content was observed under anaerobic conditions at 4 °C.

#### 3.2.5. Beta-Caryophyllene

**Figure 7:**
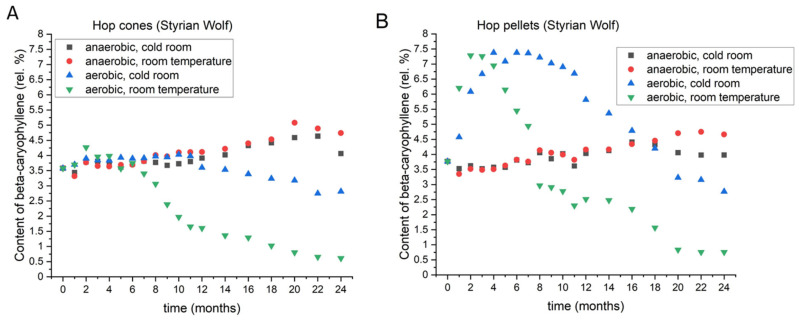
Changes in the content of beta-caryophyllene in Styrian Wolf cones (**A**) and pellets (**B**) during two-year period, under different storage conditions.

Beta-caryophyllene is one of the major sesquiterpenes found in hops. Although it has a very low solubility, some of it still ends up in beer in its native form. It delivers spicy and woody tones into beer [20,23]. Figure 7 presents the changes in beta-caryophyllene in the Styrian Wolf cones (A) and pellets (B). Data for all varieties are shown in Supplementary Table S5. The noticeable changes started around Month 8; prior to that, the differences between different storage conditions were not large. After 8 months, the cones stored at room temperature and aerobic conditions started to lose beta-caryophyllene, and at the end of the experiment, in all varieties, the levels were the lowest under those conditions. The highest rise was observed in cones stored under anaerobic conditions at room temperature. Levels also rose in the cones stored under anaerobic conditions at 4 °C, but not as much as in anaerobic conditions at room temperature. The dynamics of the changes in cones stored under aerobic conditions at 4 °C were more similar to those observed for hops stored under anaerobic conditions, but the in beta-caryophyllene was smaller. In the pellets, the variety once again played a very important role in the dynamics of losses and increases in beta-caryophyllene during the 2 years of storage. In Celeia and Styrian Wolf, the highest levels occurred under anaerobic conditions at room temperature. In Aurora, Bobek, and Savinjski Golding, the levels were highest under anaerobic conditions at low temperatures.

In all varieties, pellets showed the highest levels within 4 months if stored under aerobic conditions, no matter the temperature. Subsequently, the pellets stored at room temperature started to lose beta-caryophyllene, and those stored at 4 °C either retained their levels or lost a much smaller amount compared to pellets at room temperature. Once again, the pellets were the most stable if stored at 4 °C with no access to oxygen.

#### 3.2.6. Alpha-Humulene

**Figure 8:**
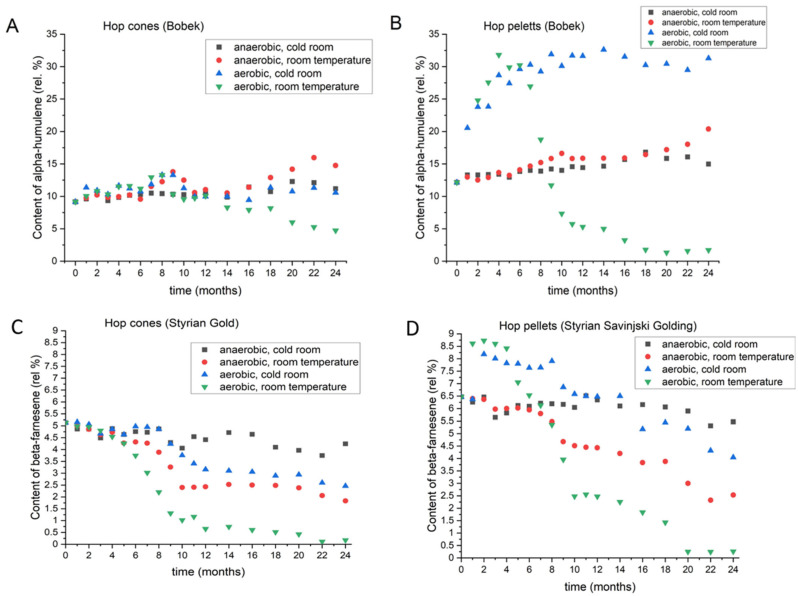
Changes in the content of alpha-humulene in Bobek cones (**A**) and pellets (**B**) and changes in the content of beta-farnesene in Styrian Golding cones (**C**) and Savinjski Golding pellets (**D**) during two-year period, under different storage conditions.

Like beta-caryophyllene, alpha-humulene delivers spicy and woody notes to beer [20]. Figure 8 shows the changes in alpha-humulene levels in Bobek cones (A) and pellets (B); Supplementary Table S6 shows the data for the other varieties. In the first few months, the changes in alpha-humulene in all the varieties followed the same dynamics, and no substantial changes were noted between the different storage conditions. After 2 years, the highest levels were achieved under anaerobic conditions at room temperature. In all varieties, the alpha-humulene content fluctuated. In Bobek, Styrian Gold, and Styrian Wolf, the smallest change after two years occurred under aerobic conditions at low temperature, whereas in Celeia and Aurora, anaerobic conditions at 4 °C caused the smallest deviation from the original value. Under aerobic conditions and at room temperature, a small rise was observed before the decline. At the end of 2 years of storage, all varieties had lost the highest amount of alpha-humulene under those storage conditions. In the pellets stored under aerobic conditions at room temperature, all varieties, except Aurora, lost alpha-humulene. In Aurora, the decline was observed much later than in the other varieties. Under aerobic conditions, regardless of the temperature, a substantial rise in alpha-humulene was observed, followed by a decline that was slower at 4 °C than at room temperature. The levels of alpha-humulene in pellets stored under anaerobic conditions followed the same pattern; however, the pellets stored at room temperature had a slightly higher alpha-humulene content.

#### 3.2.7. Beta-Farnesene

Beta-farnesene, which belongs to the group of acyclic sesquiterpenes, is only found in some hop varieties. It has a woody, herbal, and citrus aroma. It is present in all Slovenian varieties, in Czech Saaz, in German Tettnanger, and in several American varieties, but is completely absent in others, such as Hallertauer Mittelfrüh. If present, the concentration of beta-farnesene could be as much as 20% [24,25]. Figure 8 shows the changes in beta-farnesene in hop cones of Styrian Gold (C) and in hop pellets of Savinjski Golding (D); Supplementary Table S7 shows the data for the other varieties. Minor changes occurred in the hop cones of all varieties when they were stored at 4 °C, with no access to oxygen. The initial and the final contents were less than 1% apart. The dynamics of the changes in beta-farnesene were very similar under anaerobic conditions at room temperature and under aerobic conditions at 4 °C. However, at the end of 2 years of storage, only Bobek and Styrian Wolf lost more beta-farnesene if stored under anaerobic conditions at room temperature than if stored under aerobic conditions at 4 °C. The cones stored at room temperature with access to oxygen lost the majority (more than 90%) of their beta-farnesene after two years, with most of it lost in the initial 10 months. In the pellets, the dynamics of changes followed the pattern seen for alpha-humulene and beta-caryophyllene when stored under aerobic conditions but not without access to oxygen. All varieties showed a loss of beta-farnesene, but the highest concentrations were retained under anaerobic conditions at 4 °C. In the initial 6–11 months, the levels were similar in the absence of oxygen, regardless of the temperature. Subsequently, the pellets stored at room temperature lost higher amounts of beta-farnesene. Under aerobic conditions, the levels of beta-farnesene rose in the initial months, but after some time (3–8 months, depending on the variety), the pellets started to lose beta-farnesene. Under room temperature conditions, the final concentration in all varieties was below 1%.

#### 3.2.8. Caryophyllene Oxide

**Figure 9:**
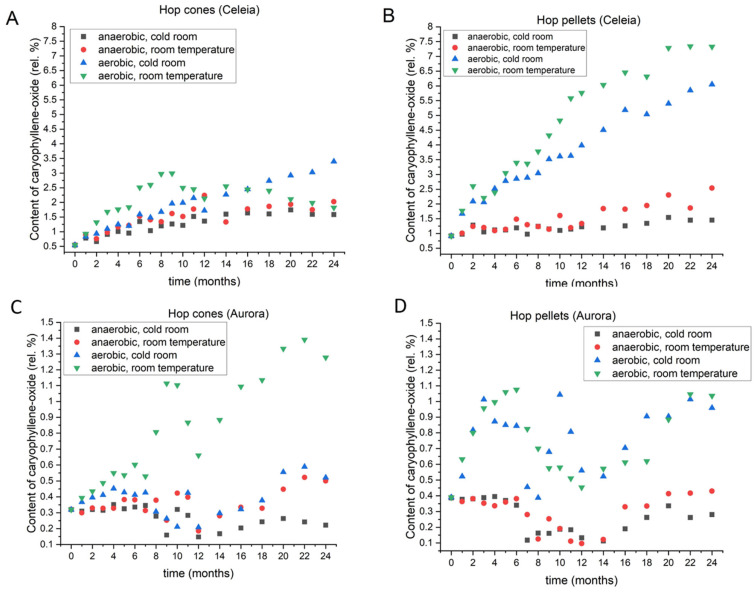
Changes in the content of caryophyllene-oxide in Celeia cones (**A**) and pellets (**B**) and Aurora cones (**C**) and pellets (**D**) during two-year period, under different storage conditions.

Caryophyllene oxide is the major oxidation product of beta-caryophyllene oxide. It is found in hop oil but rarely in beer, as it undergoes hydrolysis that results in several products. Of these, the major one is clovane diol, which has an aroma described as spicy [26]. Figure 9 shows the caryophyllene oxide contents in Celeia and Aurora, respectively. Data for the other varieties are found in Supplementary Table S8. In Celeia, Bobek, and Styrian Gold cones stored under aerobic conditions at room temperature, the level of caryophyllene oxide rose until 9–12 months, followed by a decrease. Under the same conditions, the levels in Aurora and Styrian Wolf rose and then started to decrease, followed by another rise. Therefore, among all the tested storage conditions, the highest amount of beta-caryophyllene was achieved in Aurora and Styrian Wolf under aerobic conditions at room temperature. By contrast, in Celeia, Bobek, and Styrian Gold, the highest levels were achieved if cones were stored at 4 °C, with access to oxygen. Once again, the smallest change was observed if cones were stored at 4 °C with no access to oxygen.

The dynamics of changes in pellets stored at 4 °C under anaerobic conditions were similar to those of the cones. In Celeia and Savinjski Golding pellets, the caryophyllene oxide content rose appreciably throughout the whole experimental period under anaerobic conditions at room temperature. Bobek pellets showed a slight decrease after 18 months but retained the highest levels at the end of 2 years compared to the other storage conditions. Styrian Wolf pellets showed some surprising fluctuations in caryophyllene oxide. This is the only variety among pellets that have the highest content of caryophyllene oxide under aerobic conditions at 4 °C. Styrian Wolf and Bobek showed only minor differences when the pellets were stored at different temperatures under anaerobic conditions, whereas Celeia, Aurora, and Savinjski Golding had slightly higher amounts of caryophyllene when stored at room temperature.

### 3.3. Statistical Comparison of Samples

**Figure 10:**
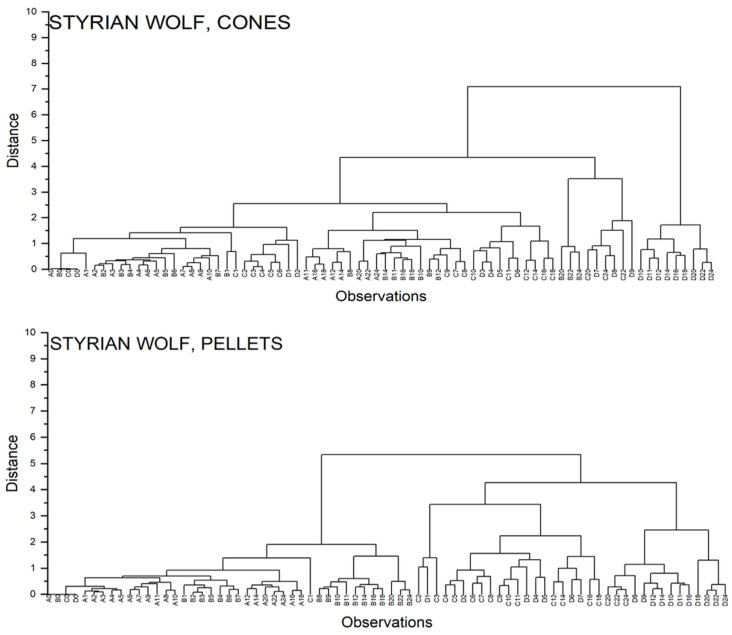
Dendrograms for Styrian Wolf cones and pellets.

Figure 10 presents the dendrogram for Styrian Wolf cones and pellets. Dendrograms for other varieties are found in the Supplementary Data (Figures S1–S8). Labeling of samples is composed of letters (A–D) and numbers, with letters labeling the conditions and numbers labeling the month of the storage. Letter A stands for anaerobic conditions in a cold room, Letter B for anaerobic conditions at room temperature, letter C for aerobic conditions in a cold room, and Letter D for aerobic conditions at room temperature.

In the case of hop cones, the biggest difference is spotted between samples stored under aerobic conditions and room temperature, from Months 9 to 14 (variety dependent), and other samples. In the next cluster, samples stored under Conditions B–D are grouped, with samples B older than 18 months (except Styrian Gold 10 months), samples C older than 16 months (except Aurora, where Group C belongs to the other cluster), and samples D older than 6 months. In the initial months, the clustering is more variety dependent, but noticeable differences are seen between various storage conditions. Taking a close look into Styrian Wolf shows no significant differences between anaerobic conditions in a cold room vs. room temperature in the first 6 months, whereas in Celeia, the differences are seen already after the second month. Samples stored under anaerobic conditions and in a cold room after two years are similar to ones stored for one year or even less under inappropriate storage conditions. In the hop pellets, it is nicely seen that the most destructive factor is oxygen presence, since samples are clustered into two groups: anaerobic and aerobic ones. The only exceptions are Aurora and Savinjski Golding pellets at 1 month of storage. Inside the anaerobic group, we have further clustering of samples stored under Condition B, older than 8 months (9–11, variety dependent), and the rest of the anaerobic samples. In the initial months, the differences between anaerobic samples are more variety dependent. In Aurora, the differences are seen only after half a year, whereas in Styrian Wolf, Celeia, and Bobek, the importance of storage conditions is seen already after the first/second month.

## 4. Discussion

Hop essential oils are composed of numerous compounds, of which the majority are volatile, so it would be expected that for proper storage, maintaining low temperatures to prevent evaporation would be enough. However, the results from our study show that another important factor is access to oxygen. In all varieties, the amount of oil lost was higher if oxygen was accessible.

Based on HCA, the samples stored at low temperatures and anaerobic conditions had the most similar composition to the original one for the longest period of time. The statistical differences between samples also show that in the initial months, the amount of changes in hop oil content and changes in oil composition are more variety dependent (especially in cones), but after, the storage conditions take the main role. Besides that, regardless of the storage conditions, some varieties lost a higher amount of oil, which proves that some varieties are inherently more stable than others. The majority of hop is crushed into pellets, since the pellets take up much less space. Another reason is the stability of pellets, since they are known to be more stable than hop cones. The higher stability is known to be the consequence of the thin film formed around the pellets, to prevent oxidation and evaporation. However, from cluster diagrams and other results, it is seen that this is true only if no access to oxygen is allowed. In the process of pelleting, the hop cones are crushed into pellets, causing cracks in lupulin glands, which contain essential oil. These cracks leave the oil more accessible to oxygen and allow a higher evaporation rate. As a consequence, the hop cones are more stable in aerobic conditions than pellets. On the contrary, the pellets performed better when anaerobic conditions were investigated. Therefore, pellets are a better option for storage, but only if anaerobic conditions are enabled. However, after 8 months, the pellets stored under anaerobic conditions and room temperature are more similar to the 2-year-old pellets stored under anaerobic conditions and in a cold room than to the original one. Myrcene, linalool, beta-caryophyllene, alpha-humulene, and beta-farnesene are the most frequent describers of the profile of hop oil. The differences in the dynamics of changes within the same variety and form indicate that during long-time storage, not only does evaporation of compounds occur, but some other biotransformation also takes place. The most abundant compound found in all varieties is myrcene. Under aerobic conditions and at room temperature, all varieties lost almost all of it, suggesting that lack of myrcene is a good indicator for old and improperly stored hops. The decline in myrcene content is observable in all storage conditions, all varieties, and all forms, meaning that no formation of myrcene occurs after harvesting. On the contrary, from myrcene, many hydration and oxidation products evolve, such as linalool and geraniol. The content of linalool increases until a sufficient amount of myrcene is still available for biotransformation. The levels of geraniol do not increase so strictly, probably due to the fact that linalool can also be formed from geraniol. Alpha-humulene and beta-caryophyllene, both sesquiterpenes, have very similar dynamics of changes. In the initial months, only minor changes occur, which shows that they are less volatile and less prone to oxidation than some other compounds. Another biotransformation that takes place in lupulin glands is the oxidation of beta-caryophyllene into caryophyllene oxide. As expected, the dynamics of the changes in the concentrations of caryophyllene oxide were the inverse of those for beta-caryophyllene, but they were not precisely the same, especially in the initial months, when the levels of beta-caryophyllene did not change much, indicating that some other conversions were taking place at the same time. When comparing cones and pellets in alpha-pinene, alpha-humulene, beta-caryophyllene, and caryophyllene oxide, it is seen that the dynamics of the changes within the same variety, compound, and condition differ from another. This points to the fact that with pelletizing, not only is the shape of the hop changed, but some other more complex changes are made. A possible explanation for this might be the temperature, which increases during the process and might have an impact on the enzymes responsible for biotransformation. A further study with more focus on the differences between cones and pellets is therefore suggested.

## 5. Conclusions

Changes in hop oil content and in eight compounds in hop oil after storage under different conditions were evaluated. To our knowledge, this is the first study evaluating the difference in the relative amount of alpha-pinene, myrcene, linalool, geraniol, alpha-humulene, beta-caryophyllene, beta-farnesene, and caryophyllene oxide under different storage conditions during long-time storage. Together, the present findings confirm that for maintaining the highest possible amount of hop oil and original composition, many factors such as storage conditions, variety, and form of hop have to be taken into consideration. Storage at low temperatures and anaerobic conditions is the best. Low levels of myrcene turned out to be a great indicator of improper storage conditions. The results also provide evidence that with hop pelleting, not only is the shape of hop changed, but some yet unexplained changes occur. Based on our results, the expiration dates for hop should be more individually formed, based on hop variety, since the storage stability is hugely variety dependent. The concept of our study could be interesting for hop merchants, in order to determine a more accurate expiry date.

## Figures and Tables

**Table 1:** Information about hop material.

Variety	Cones	Pellets	Supplier
Celeia	x	x	Hmezad Exim d.d.
Aurora	x	x	Hmezad Exim d.d.
Bobek	x	x	Farm Bosnar for hop cones and Hmezad Exim d.d. for pellets
Styrian Gold	x		Hmezad Exim d.d.
Savinjski golding		x	Hmezad Exim d.d.
Styrian Wolf	x	x	Farm Bizjak for hop cones and Hmezad Exim d.d. for hop pellets

**Table 2:** Loss of the hop essential oil in% with standard deviations, after two years of storage under different conditions.

Storage Conditions	Variety	Shape of Hops	Loss of Oil [%] [Mean ± SD]
Anaerobic conditions, cold room	Celeia	cones	63.3 ± 0.8
	Celeia	pellets	53.3 ± 0.7
	Aurora	cones	35.6 ± 0.4
	Aurora	pellets	31.0 ± 0.4
	Bobek	cones	54.8 ± 0.7
	Bobek	pellets	46.8 ± 0.6
	Styrian Gold	cones	45.0 ± 0.6
	Savinjski Golding	pellets	44.0 ± 0.5
	Styrian Wolf	cones	49.6 ± 0.6
	Styrian Wolf	pellets	31.2 ± 0.4
Anaerobic conditions, room temperature	Celeia	cones	66.4 ± 0.8
	Celeia	pellets	60.7 ± 0.7
	Aurora	cones	63.9 ± 0.8
	Aurora	pellets	46.1 ± 0.6
	Bobek	cones	71.9 ± 0.9
	Bobek	pellets	56.6 ± 0.7
	Styrian Gold	cones	66.4 ± 0.8
	Savinjski Golding	pellets	66.7 ± 0.8
	Styrian Wolf	cones	65.4 ± 0.8
	Styrian Wolf	pellets	47.9 ± 0.6
Aerobic conditions, cold room	Celeia	cones	64.1 ± 0.8
	Celeia	pellets	73.8 ± 0.9
	Aurora	cones	55.0 ± 0.7
	Aurora	pellets	73.7 ± 0.9
	Bobek	cones	70.6 ± 0.9
	Bobek	pellets	85.7 ± 1.1
	Styrian Gold	cones	60.9 ± 0.7
	Savinjski Golding	pellets	70.7 ± 0.9
	Styrian Wolf	cones	72.0 ± 0.9
	Styrian Wolf	pellets	87.8 ± 1.1
Aerobic conditions, room temperature	Celeia	cones	74.2 ± 0.9
	Celeia	pellets	84.1 ± 1.0
	Aurora	cones	79.3 ± 1.0
	Aurora	pellets	85.5 ± 1.1
	Bobek	cones	84.6 ± 1.0
	Bobek	pellets	88.4 ± 1.1
	Styrian Gold	cones	71.9 ± 0.9
	Savinjski Golding	pellets	86.7 ± 1.0
	Styrian Wolf	cones	79.4 ± 1.0
	Styrian Wolf	pellets	91.1 ± 1.1

## Supplementary Materials

The following supporting information can be downloaded at: https://www.mdpi.com/article/10.3390/foods11193089/s1, Table S1: Changes in alpha-pinen content in all varieties for all storage conditions; Table S2: Changes in myrcene content in all varieties for all storage conditions; Table S3: Changes in linalool content in all varieties for all storage conditions; Table S4: Changes in geraniol content in all varieties for all storage conditions; Table S5: Changes in beta-caryophyllene content in all varieties for all storage conditions; Table S6: Changes in alpha-humulene content in all varieties for all storage conditions; Table S7: Changes in beta-farnesene content in all varieties for all storage conditions; Table S8: Changes in caryophyllene-oxide content in all varieties for all storage conditions, Figure S1: Dendrogram for cones of Celeia; Figure S2: Dendrogram for pellets of Celeia; Figure S3: Dendrogram for cones of Aurora; Figure S4: Dendrogram for pellets of Aurora; Figure S5: Dendrogram for cones of Bobek; Figure S6: Dendrogram for pellets of Bobek; Figure S7: Dendrogram for cones of Styrian Gold; Figure S8: Dendrogram for pellets of Savinjski Golding.
