# Supplementary material for: Changes in Hop (Humulus lupulus L.) Oil Content and Composition during Long-Term Storage under Different Conditions
Source: Foods. 2022 Oct 5;11(19):3089. doi: 10.3390/foods11193089 (PMC9563567; doi:10.3390/foods11193089)
Supplement: Supplementary file 1 [file foods-11-03089-s001.zip › Supplementary tables and figures.pdf]

Supplementary Table S1. Changes in alpha-pinen content in all varieties for all storage conditions.

Variety/form	time [months] / conditions	0	1	2	3	4	5	6	7	8	9	10	11	12	14	16	18	20	22	24
		Content of alpha-pinene [rel. %]																		
Celeia cones	anaerobic, cold room	0,08	0,08	0,09	0,10	0,10	0,10	0,09	0,10	0,11	0,11	0,11	0,12	0,12	0,12	0,12	0,11	0,12	0,12	0,14
	anaerobic, room temperature	0,08	0,09	0,08	0,10	0,10	0,11	0,11	0,13	0,13	0,17	0,20	0,19	0,20	0,20	0,20	0,16	0,26	0,35	0,39
	aerobic, cold room	0,08	0,08	0,08	0,09	0,11	0,11	0,10	0,11	0,12	0,11	0,12	0,12	0,12	0,13	0,15	0,15	0,16	0,19	0,23
	aerobic, room temperature	0,08	0,08	0,08	0,10	0,12	0,13	0,14	0,16	0,22	0,26	0,31	0,40	0,41	0,42	0,42	0,43	0,44	0,46	0,62
Celeia pellets	anaerobic, cold room	0,05	0,06	0,06	0,06	0,06	0,06	0,06	0,07	0,06	0,06	0,06	0,06	0,06	0,06	0,07	0,06	0,06	0,07	0,07
	anaerobic, room temperature	0,05	0,05	0,06	0,06	0,07	0,07	0,07	0,07	0,07	0,08	0,08	0,09	0,09	0,10	0,09	0,09	0,08	0,09	0,10
	aerobic, cold room	0,05	0,02	0,02	0,02	0,02	0,02	0,01	0,02	0,02	0,01	0,01	0,01	0,01	0,01	0,01	0,01	0,01	0,01	0,01
	aerobic, room temperature	0,05	0,02	0,01	0,01	0,01	0,00	0,00	0,00	0,00	0,00	0,00	0,00	0,01	0,01	0,00	0,01	0,00	0,01	0,01
Aurora cones	anaerobic, cold room	0,08	0,09	0,09	0,09	0,09	0,09	0,09	0,11	0,08	0,09	0,09	0,09	0,10	0,11	0,12	0,11	0,11	0,13	0,12
	anaerobic, room temperature	0,08	0,10	0,10	0,09	0,10	0,10	0,10	0,08	0,11	0,13	0,14	0,15	0,17	0,17	0,17	0,17	0,22	0,29	0,29
	aerobic, cold room	0,08	0,11	0,09	0,09	0,09	0,09	0,10	0,09	0,09	0,09	0,11	0,11	0,11	0,12	0,15	0,13	0,12	0,14	0,16
	aerobic, room temperature	0,08	0,11	0,09	0,10	0,10	0,11	0,12	0,14	0,19	0,35	0,45	0,40	0,46	0,58	0,60	0,55	0,62	0,77	0,79
Aurora pellets	anaerobic, cold room	0,08	0,09	0,10	0,10	0,10	0,12	0,10	0,10	0,10	0,10	0,10	0,11	0,11	0,12	0,11	0,13	0,12	0,13	0,14
	anaerobic, room temperature	0,08	0,09	0,10	0,11	0,11	0,13	0,11	0,14	0,15	0,16	0,17	0,19	0,20	0,20	0,21	0,20	0,22	0,24	0,26
	aerobic, cold room	0,08	0,07	0,05	0,04	0,04	0,03	0,04	0,03	0,03	0,02	0,02	0,02	0,02	0,02	0,01	0,03	0,02	0,02	0,02
	aerobic, room temperature	0,08	0,05	0,02	0,03	0,01	0,00	0,01	0,00	0,01	0,01	0,01	0,01	0,01	0,02	0,02	0,02	0,01	0,02	0,02
Bobek cones	anaerobic, cold room	0,10	0,10	0,11	0,11	0,12	0,11	0,12	0,11	0,12	0,13	0,14	0,13	0,13	0,14	0,15	0,16	0,16	0,18	0,16
	anaerobic, room temperature	0,10	0,10	0,11	0,11	0,12	0,12	0,13	0,13	0,13	0,15	0,25	0,24	0,23	0,28	0,30	0,31	0,37	0,46	0,51
	aerobic, cold room	0,10	0,10	0,11	0,12	0,12	0,15	0,14	0,13	0,11	0,12	0,18	0,19	0,20	0,21	0,26	0,24	0,27	0,31	0,31
	aerobic, room temperature	0,10	0,10	0,10	0,11	0,13	0,18	0,20	0,26	0,40	0,53	0,74	0,76	0,81	0,84	0,84	0,83	0,83	0,97	1,03
Bobek pellets	anaerobic, cold room	0,07	0,08	0,09	0,08	0,08	0,09	0,08	0,08	0,08	0,09	0,08	0,09	0,08	0,09	0,09	0,09	0,09	0,09	0,09
	anaerobic, room temperature	0,07	0,08	0,08	0,09	0,09	0,09	0,10	0,10	0,11	0,13	0,12	0,14	0,13	0,13	0,13	0,14	0,14	0,17	0,19
	aerobic, cold room	0,07	0,06	0,05	0,05	0,04	0,04	0,03	0,02	0,02	0,01	0,02	0,02	0,02	0,02	0,01	0,01	0,01	0,02	0,02
	aerobic, room temperature	0,07	0,05	0,03	0,02	0,02	0,01	0,01	0,01	0,01	0,02	0,02	0,02	0,03	0,03	0,03	0,03	0,02	0,04	0,03
Styrian Gold cones	anaerobic, cold room	0,08	0,09	0,08	0,09	0,08	0,09	0,08	0,08	0,07	0,09	0,09	0,09	0,08	0,07	0,08	0,10	0,10	0,11	0,10
	anaerobic, room temperature	0,08	0,08	0,08	0,09	0,08	0,10	0,10	0,11	0,13	0,14	0,19	0,13	0,14	0,21	0,15	0,18	0,21	0,24	0,34
	aerobic, cold room	0,08	0,07	0,07	0,09	0,08	0,09	0,08	0,08	0,07	0,08	0,10	0,09	0,09	0,13	0,11	0,11	0,12	0,13	0,15
	aerobic, room temperature	0,08	0,08	0,08	0,08	0,09	0,10	0,12	0,19	0,28	0,27	0,28	0,29	0,28	0,27	0,27	0,31	0,46	0,55	0,48
Savinjski golding pellets	anaerobic, cold room	0,05	0,05	0,05	0,06	0,06	0,06	0,06	0,06	0,06	0,06	0,05	0,05	0,06	0,06	0,05	0,05	0,05	0,05	0,07
	anaerobic, room temperature	0,05	0,05	0,05	0,06	0,06	0,06	0,07	0,07	0,07	0,07	0,08	0,07	0,09	0,08	0,09	0,08	0,08	0,08	0,11
	aerobic, cold room	0,05	0,05	0,02	0,02	0,02	0,00	0,02	0,01	0,01	0,01	0,01	0,01	0,01	0,01	0,00	0,01	0,00	0,01	0,01
	aerobic, room temperature	0,05	0,01	0,01	0,01	0,00	0,00	0,00	0,00	0,00	0,00	0,00	0,00	0,00	0,00	0,01	0,01	0,01	0,00	0,00
Styrian Wolf cones	anaerobic, cold room	0,11	0,11	0,12	0,10	0,11	0,11	0,11	0,12	0,13	0,13	0,13	0,12	0,12	0,14	0,14	0,14	0,16	0,14	0,16
	anaerobic, room temperature	0,11	0,22	0,13	0,13	0,14	0,12	0,02	0,17	0,19	0,24	0,33	0,27	0,30	0,30	0,33	0,29	0,43	0,47	0,55
	aerobic, cold room	0,11	0,12	0,12	0,13	0,13	0,15	0,15	0,16	0,16	0,17	0,18	0,22	0,20	0,25	0,26	0,29	0,36	0,34	0,43
	aerobic, room temperature	0,11	0,14	0,15	0,15	0,19	0,29	0,26	0,37	0,45	0,66	0,78	0,83	0,88	1,04	0,89	0,95	0,94	0,92	0,96
Strian Wolf pellets	anaerobic, cold room	0,10	0,10	0,10	0,11	0,10	0,10	0,11	0,11	0,10	0,12	0,11	0,12	0,13	0,13	0,12	0,13	0,14	0,15	0,15
	anaerobic, room temperature	0,10	0,12	0,13	0,13	0,16	0,14	0,15	0,16	0,18	0,18	0,20	0,21	0,21	0,22	0,22	0,22	0,25	0,29	0,28
	aerobic, cold room	0,10	0,09	0,07	0,06	0,04	0,05	0,04	0,05	0,03	0,03	0,03	0,04	0,05	0,05	0,03	0,02	0,03	0,03	0,03
	aerobic, room temperature	0,10	0,05	0,02	0,01	0,01	0,02	0,02	0,03	0,01	0,03	0,03	0,04	0,04	0,04	0,03	0,03	0,03	0,04	0,05

Supplementary Table S2. Changes in myrcene content in all varieties for all storage conditions.

Variety/form	conditions \ time [months]	0	1	2	3	4	5	6	7	8	9	10	11	12	14	16	18	20	22	24
		Content of myrcene [rel. %]																		
Celeia cones	anaerobic, cold room	48,44	47,44	49,29	46,69	45,95	45,45	41,40	44,39	41,10	40,43	42,01	39,88	41,01	39,80	36,42	34,48	34,73	34,75	36,09
	anaerobic, room temperature	48,44	45,73	46,78	44,47	43,12	41,18	38,16	37,76	35,64	33,36	32,00	31,25	30,03	27,88	26,43	20,86	11,11	11,48	12,78
	aerobic, cold room	48,44	44,76	44,25	43,92	41,45	43,45	38,74	40,65	38,65	34,88	34,56	36,53	34,42	33,38	31,55	26,09	18,50	22,22	20,74
	aerobic, room temperature	48,44	42,54	38,28	37,10	36,39	35,17	28,24	24,67	18,78	11,35	5,63	4,16	3,99	3,00	2,98	2,41	1,79	1,51	2,14
Celeia pellets	anaerobic, cold room	39,13	38,40	38,19	37,79	37,04	36,47	34,68	34,60	34,50	34,70	34,95	34,80	34,31	29,42	28,40	27,39	28,12	27,55	30,32
	anaerobic, room temperature	39,13	38,08	37,08	35,74	37,26	36,08	32,35	33,07	31,21	29,77	24,37	25,00	25,11	20,37	19,78	18,06	13,46	15,08	8,72
	aerobic, cold room	39,13	19,93	9,65	6,32	2,11	6,17	3,28	5,87	4,70	3,65	2,94	3,34	4,27	3,22	2,14	1,73	1,64	1,52	1,85
	aerobic, room temperature	39,13	13,85	5,98	2,90	4,42	1,46	1,28	0,94	0,94	0,53	0,39	0,91	1,37	0,73	0,52	0,50	0,33	0,23	0,09
Aurora cones	anaerobic, cold room	57,54	58,11	57,70	57,71	56,00	55,56	57,73	52,73	52,38	53,86	54,13	56,03	53,15	52,18	55,86	50,64	50,43	53,41	54,71
	anaerobic, room temperature	57,54	57,87	57,20	57,73	56,69	55,09	56,67	55,95	48,74	49,26	45,02	46,99	51,05	48,90	44,78	45,01	36,28	30,88	29,52
	aerobic, cold room	57,54	52,92	53,02	53,47	52,28	51,15	52,36	49,63	49,44	45,34	50,74	49,49	52,42	48,70	45,72	43,24	38,15	38,24	36,67
	aerobic, room temperature	57,54	51,93	50,47	52,02	48,88	48,44	46,94	38,51	27,44	17,01	10,14	6,88	8,35	6,41	4,87	2,96	1,78	1,52	1,49
Aurora pellets	anaerobic, cold room	53,44	51,80	51,99	52,20	51,01	48,58	52,20	51,33	49,39	49,15	47,97	50,53	48,64	47,51	42,16	43,02	43,02	48,44	47,80
	anaerobic, room temperature	53,44	51,38	52,50	53,01	52,75	50,69	50,66	50,07	48,24	46,15	43,89	47,25	49,17	45,57	43,53	42,90	37,57	37,79	35,83
	aerobic, cold room	53,44	39,82	22,03	12,77	13,07	6,61	10,11	7,62	7,59	4,17	3,97	5,44	4,90	3,19	2,23	2,21	1,89	1,97	4,96
	aerobic, room temperature	53,44	30,14	7,55	4,68	2,91	1,67	1,74	1,46	1,02	0,78	1,58	1,95	1,25	0,70	0,54	0,49	0,57	0,17	0,16
Bobek cones	anaerobic, cold room	68,36	66,26	63,69	65,45	63,53	62,84	61,45	60,59	60,15	60,22	60,31	61,97	60,27	62,31	56,32	57,47	51,50	51,59	57,26
	anaerobic, room temperature	68,36	66,16	64,04	63,51	63,54	61,12	63,01	57,29	51,12	46,15	45,09	56,59	54,16	51,96	48,78	46,26	34,35	29,09	28,64
	aerobic, cold room	68,36	60,10	61,24	61,87	55,90	55,96	57,52	52,30	49,55	47,12	51,55	54,36	52,30	51,31	48,45	40,26	38,09	28,08	30,93
	aerobic, room temperature	68,36	64,91	60,98	61,79	55,65	52,75	49,57	36,18	22,15	13,09	11,10	11,60	10,39	10,49	9,00	5,30	4,54	4,22	4,08
Bobek pellets	anaerobic, cold room	56,95	56,83	56,12	57,73	57,37	59,10	56,80	56,19	56,64	56,61	56,46	55,58	55,26	55,54	53,02	47,91	51,38	51,01	54,31
	anaerobic, room temperature	56,95	58,22	59,76	58,90	56,89	58,75	57,09	54,79	53,61	53,07	51,63	52,71	51,98	51,11	52,84	51,43	49,71	47,02	39,56
	aerobic, cold room	56,95	32,96	22,84	23,28	10,25	13,42	8,28	6,62	4,58	4,48	5,64	4,72	3,65	2,91	2,19	2,00	1,78	2,38	2,16
	aerobic, room temperature	56,95	25,00	20,69	11,22	1,94	2,83	1,46	1,02	0,66	1,27	0,72	0,55	0,45	0,43	0,34	0,33	0,23	0,88	0,61
Styrian Gold cones	anaerobic, cold room	46,70	43,37	43,57	46,67	44,33	44,72	40,60	39,10	38,06	37,63	37,50	37,16	25,91	29,30	29,88	32,85	30,86	34,56	36,23
	anaerobic, room temperature	46,70	40,53	42,53	44,58	42,70	43,14	37,38	37,92	32,37	29,48	23,87	20,19	15,46	26,98	29,48	18,29	18,14	15,39	16,97
	aerobic, cold room	46,70	39,54	39,43	44,24	41,54	43,71	37,41	39,11	34,15	34,10	34,14	30,12	23,06	31,69	27,17	22,94	23,79	19,92	27,70
	aerobic, room temperature	46,70	38,89	40,55	40,73	40,94	37,59	30,59	27,62	17,45	9,52	7,53	5,87	3,97	5,33	3,80	3,27	2,28	2,24	1,36
Savinjski golding pellets	anaerobic, cold room	32,03	33,90	32,93	34,27	33,53	32,61	34,45	30,46	31,46	30,17	25,37	24,24	26,45	26,25	24,10	24,63	20,39	19,30	26,07
	anaerobic, room temperature	32,03	31,44	32,56	32,60	31,12	30,53	31,55	28,11	26,57	22,57	18,22	19,73	21,16	19,54	17,31	16,69	12,85	8,78	9,97
	aerobic, cold room	32,03	33,77	12,97	7,69	5,35	2,31	5,92	4,82	3,77	3,27	2,76	3,99	3,92	2,24	1,64	1,88	1,36	1,34	1,75
	aerobic, room temperature	32,03	5,32	4,84	2,84	1,72	1,31	1,42	1,12	0,78	0,58	1,63	1,30	0,86	0,75	0,63	0,47	0,38	0,55	0,04
Styrian Wolf cones	anaerobic, cold room	60,78	60,38	56,78	56,20	57,49	58,26	57,75	55,85	56,70	57,46	56,45	48,70	55,33	54,06	49,18	48,77	43,96	42,70	38,35
	anaerobic, room temperature	60,78	59,62	56,73	57,09	56,43	56,00	55,62	54,14	51,53	48,48	44,78	40,96	46,81	46,51	39,66	41,04	27,89	22,24	24,47
	aerobic, cold room	60,78	58,88	53,36	52,67	51,89	49,95	49,20	46,95	43,93	43,49	36,74	33,98	42,10	38,79	34,45	30,36	23,18	17,49	17,00
	aerobic, room temperature	60,78	54,03	45,60	44,35	41,82	39,84	34,91	25,76	17,76	8,67	5,71	4,56	4,78	3,45	2,90	2,18	1,32	1,10	1,09
Strian Wolf pellets	anaerobic, cold room	53,47	56,89	55,55	56,43	55,09	56,10	53,76	55,14	50,96	53,37	51,53	56,33	50,45	51,79	46,64	47,76	48,74	52,39	51,54
	anaerobic, room temperature	53,47	58,25	56,42	56,67	55,46	55,39	54,05	54,11	51,26	49,79	50,66	53,22	48,20	50,11	47,31	45,67	39,57	41,45	41,15
	aerobic, cold room	53,47	44,00	26,35	16,79	4,63	7,85	4,72	4,08	2,89	3,37	3,85	3,35	2,14	2,07	1,99	1,39	0,41	1,08	1,47
	aerobic, room temperature	53,47	44,00	26,35	16,79	4,63	7,85	4,72	4,08	2,89	3,37	3,85	3,35	2,14	0,63	0,51	0,39	0,30	0,27	0,21

Supplementary Table S3. Changes in linalool content in all varieties for all storage conditions.

Variety/form	time [months] / conditions	0	1	2	3	4	5	6	7	8	9	10	11	12	14	16	18	20	22	24
		Content of linalool [rel. %]																		
Celeia cones	anaerobic, cold room	0,89	0,90	0,95	0,97	1,02	0,97	1,06	1,06	1,07	1,11	1,10	1,17	1,22	1,23	1,27	1,21	1,33	1,15	1,18
	anaerobic, room temperature	0,89	1,01	0,98	1,02	1,10	1,05	1,20	1,17	1,23	1,35	1,45	1,48	1,25	1,65	1,67	1,57	2,00	1,96	1,87
	aerobic, cold room	0,89	1,05	1,02	1,00	1,04	1,02	1,14	1,16	1,14	1,21	1,14	1,30	1,53	1,50	1,56	1,44	1,86	1,59	1,76
	aerobic, room temperature	0,89	1,09	1,16	1,30	1,24	1,21	1,64	1,45	1,62	1,64	1,68	2,29	2,41	2,22	2,12	2,07	1,81	1,99	2,13
Celeia pellets	anaerobic, cold room	1,23	1,34	1,21	1,26	1,29	1,28	1,32	1,32	1,40	1,27	1,35	1,41	1,43	1,49	1,57	1,42	1,54	1,58	1,56
	anaerobic, room temperature	1,23	1,29	1,30	1,30	1,42	1,41	1,44	1,48	1,46	1,51	1,70	1,59	1,70	2,20	2,03	1,72	2,04	1,95	2,10
	aerobic, cold room	1,23	1,62	1,70	1,75	1,69	1,42	1,57	1,69	1,62	1,60	1,61	1,72	1,73	2,00	1,57	1,34	1,28	1,22	1,38
	aerobic, room temperature	1,23	1,49	1,74	1,77	1,77	1,42	1,50	1,70	1,35	1,51	1,22	1,05	0,95	1,05	0,88	0,85	0,73	0,65	0,63
Aurora cones	anaerobic, cold room	1,15	1,22	1,33	1,45	1,43	1,39	1,41	1,67	1,81	1,48	1,47	1,60	1,43	1,56	1,41	1,54	1,54	1,66	1,52
	anaerobic, room temperature	1,15	1,28	1,32	1,43	1,48	1,52	1,55	1,43	1,82	1,75	1,78	1,92	1,91	1,94	1,92	2,04	2,24	2,53	2,61
	aerobic, cold room	1,15	1,28	1,37	1,35	1,42	1,38	1,43	1,44	1,40	1,54	1,39	1,50	1,30	1,50	1,58	1,53	1,70	1,67	1,85
	aerobic, room temperature	1,15	1,38	1,43	1,47	1,47	1,57	1,53	1,55	2,06	2,12	2,46	2,25	2,38	2,48	2,65	2,80	2,90	2,70	2,65
Aurora pellets	anaerobic, cold room	1,48	1,43	1,48	1,62	1,63	1,68	1,70	1,58	1,65	1,70	1,60	1,65	1,72	1,83	1,89	1,68	2,00	1,72	1,80
	anaerobic, room temperature	1,48	1,45	1,47	1,59	1,71	1,76	1,75	1,67	1,71	1,89	1,79	1,93	1,93	2,10	2,18	2,00	2,34	2,28	2,45
	aerobic, cold room	1,48	1,76	2,30	2,59	2,60	2,72	2,86	2,60	2,86	2,82	2,88	2,85	2,96	3,23	2,79	2,80	2,90	2,66	2,94
	aerobic, room temperature	1,48	2,11	2,60	2,68	2,90	2,50	2,80	2,63	2,82	3,03	2,92	2,59	2,59	2,59	2,63	2,38	1,64	1,54	1,39
Bobek cones	anaerobic, cold room	1,41	1,49	1,58	1,56	1,68	1,61	1,80	1,78	1,80	2,07	1,83	1,76	1,83	1,90	2,06	2,04	2,22	2,35	2,05
	anaerobic, room temperature	1,41	1,46	1,61	1,70	1,74	1,73	1,85	1,92	2,22	2,62	2,74	2,55	2,43	3,26	2,89	2,72	3,49	3,63	4,23
	aerobic, cold room	1,41	1,66	1,66	1,71	1,94	1,85	1,96	2,10	2,01	2,44	2,25	2,31	2,37	2,43	2,78	2,77	2,91	3,31	3,22
	aerobic, room temperature	1,41	1,50	1,68	1,70	2,01	1,97	2,26	3,17	3,41	3,14	3,89	4,75	4,60	4,81	4,91	4,51	4,05	4,20	4,23
Bobek pellets	anaerobic, cold room	1,58	1,47	1,69	1,57	1,54	1,61	1,60	1,60	1,58	1,53	1,69	1,65	1,66	1,68	1,65	2,00	1,84	1,79	1,75
	anaerobic, room temperature	1,58	1,48	1,58	1,63	1,60	1,67	1,72	1,78	1,85	1,81	1,83	1,93	2,05	2,00	1,95	1,97	2,03	2,32	2,66
	aerobic, cold room	1,58	2,39	2,82	2,85	3,24	3,10	3,20	3,42	2,93	3,09	3,56	3,45	3,42	3,87	2,50	2,88	2,99	2,74	2,91
	aerobic, room temperature	1,58	2,44	2,99	3,29	3,11	3,52	3,34	3,26	3,54	2,26	2,03	2,00	1,93	1,93	1,92	1,85	1,35	1,30	1,18
Styrian Gold cones	anaerobic, cold room	0,32	0,42	0,41	0,43	0,39	0,44	0,48	0,47	0,49	0,51	0,52	0,60	0,57	0,52	0,57	0,59	0,60	0,62	0,62
	anaerobic, room temperature	0,32	0,41	0,44	0,44	0,42	0,51	0,49	0,61	0,58	0,60	0,63	0,82	0,82	0,82	0,73	0,70	0,81	0,86	0,92
	aerobic, cold room	0,32	0,42	0,42	0,43	0,40	0,43	0,46	0,41	0,47	0,47	0,51	0,59	0,60	0,63	0,68	0,68	0,69	0,74	0,82
	aerobic, room temperature	0,32	0,41	0,43	0,40	0,43	0,47	0,56	0,62	0,73	0,76	0,80	0,89	0,92	0,99	0,82	0,81	0,88	0,93	0,87
Savinjski golding pellets	anaerobic, cold room	0,85	0,87	0,76	0,77	0,80	0,83	0,96	0,92	0,89	0,93	0,89	0,88	0,89	0,95	0,94	0,85	0,90	0,83	1,05
	anaerobic, room temperature	0,85	0,98	0,77	0,90	0,90	0,90	0,99	0,96	1,02	1,14	1,13	1,12	1,17	1,23	1,20	1,21	1,25	1,16	1,35
	aerobic, cold room	0,85	0,81	0,89	1,06	0,98	0,90	0,93	0,95	0,91	0,90	0,88	0,95	1,03	1,00	0,96	0,85	0,90	0,89	0,82
	aerobic, room temperature	0,85	0,87	1,03	0,98	1,02	0,81	0,99	0,88	0,79	0,78	0,77	0,76	0,65	0,44	0,38	0,56	0,50	0,30	0,33
Styrian Wolf cones	anaerobic, cold room	1,39	1,44	1,60	1,66	1,57	1,54	1,55	1,62	1,66	1,66	1,73	2,00	1,81	1,95	2,15	2,00	2,48	2,28	2,74
	anaerobic, room temperature	1,39	1,56	1,61	1,67	1,70	1,65	1,74	1,82	1,99	2,29	2,73	2,54	2,34	2,61	2,71	2,54	3,25	3,26	3,41
	aerobic, cold room	1,39	1,55	1,81	1,81	1,77	1,88	1,96	2,05	2,13	2,30	2,31	2,68	2,34	2,48	2,76	2,72	3,19	2,99	3,48
	aerobic, room temperature	1,39	1,67	2,15	2,09	2,23	2,26	2,47	2,96	3,08	3,49	3,47	3,58	3,89	4,10	3,72	4,01	3,60	3,40	3,34
Strian Wolf pellets	anaerobic, cold room	1,75	1,66	1,72	1,73	1,66	1,68	1,88	1,79	1,92	1,85	1,93	1,95	2,23	2,02	2,19	2,15	2,37	2,06	2,20
	anaerobic, room temperature	1,75	1,73	1,79	1,81	1,86	1,86	1,95	1,99	2,09	2,19	2,23	2,24	2,56	2,28	2,42	2,38	2,93	2,82	2,95
	aerobic, cold room	1,75	2,24	3,13	3,53	3,76	3,99	4,11	4,22	4,38	4,51	4,54	4,94	3,72	4,06	3,60	3,76	3,38	3,54	3,33
	aerobic, room temperature	1,75	1,36	4,05	5,40	4,36	3,84	3,74	3,27	3,25	2,77	3,08	3,16	3,29	3,07	3,00	2,30	1,73	1,82	1,49

Supplementary Table S4. Changes in geraniol content in all varieties for all storage conditions.

Variety/form	conditions \ time [months]	0	1	2	3	4	5	6	7	8	9	10	11	12	14	16	18	20	22	24
		Content of geraniol [rel. %]																		
Celeia cones	anaerobic, cold room	0,12	0,11	0,08	0,07	0,12	0,14	0,12	0,13	0,09	0,12	0,11	0,08	0,12	0,10	0,16	0,15	0,16	0,18	0,14
	anaerobic, room temperature	0,12	0,07	0,11	0,12	0,12	0,14	0,15	0,16	0,13	0,11	0,11	0,11	0,11	0,14	0,12	0,22	0,26	0,22	0,25
	aerobic, cold room	0,12	0,10	0,08	0,11	0,12	0,13	0,14	0,14	0,12	0,13	0,09	0,09	0,09	0,10	0,23	0,23	0,21	0,16	0,14
	aerobic, room temperature	0,12	0,13	0,12	0,11	0,13	0,17	0,18	0,20	0,19	0,20	0,27	0,29	0,32	0,32	0,31	0,32	0,29	0,29	0,34
Celeia pellets	anaerobic, cold room	0,13	0,12	0,10	0,09	0,13	0,12	0,13	0,13	0,12	0,11	0,11	0,11	0,12	0,17	0,16	0,15	0,14	0,12	0,16
	anaerobic, room temperature	0,13	0,12	0,12	0,11	0,13	0,14	0,15	0,15	0,14	0,13	0,16	0,17	0,18	0,22	0,21	0,20	0,24	0,25	0,26
	aerobic, cold room	0,13	0,18	0,18	0,22	0,22	0,20	0,24	0,25	0,24	0,24	0,27	0,27	0,28	0,27	0,26	0,22	0,26	0,31	0,32
	aerobic, room temperature	0,13	0,16	0,19	0,23	0,22	0,22	0,27	0,29	0,27	0,29	0,31	0,28	0,26	0,27	0,27	0,25	0,17	0,17	0,17
Aurora cones	anaerobic, cold room	0,29	0,26	0,27	0,31	0,30	0,30	0,30	0,33	0,31	0,32	0,32	0,32	0,28	0,28	0,29	0,30	0,30	0,31	0,28
	anaerobic, room temperature	0,29	0,26	0,28	0,31	0,31	0,33	0,33	0,28	0,38	0,37	0,43	0,40	0,34	0,34	0,34	0,42	0,49	0,58	0,61
	aerobic, cold room	0,29	0,29	0,31	0,33	0,36	0,35	0,35	0,34	0,34	0,35	0,35	0,35	0,28	0,30	0,33	0,39	0,43	0,43	0,46
	aerobic, room temperature	0,29	0,32	0,33	0,35	0,38	0,41	0,40	0,43	0,60	0,65	0,70	0,78	0,77	0,75	0,83	0,85	0,80	0,84	0,75
Aurora pellets	anaerobic, cold room	0,33	0,31	0,30	0,34	0,34	0,36	0,36	0,31	0,34	0,34	0,31	0,34	0,34	0,32	0,19	0,16	0,18	0,17	0,19
	anaerobic, room temperature	0,33	0,31	0,30	0,33	0,35	0,37	0,38	0,35	0,38	0,42	0,39	0,40	0,38	0,39	0,42	0,43	0,48	0,49	0,51
	aerobic, cold room	0,33	0,42	0,55	0,66	0,70	0,77	0,73	0,76	0,74	0,72	0,69	0,82	0,92	0,89	0,95	0,95	0,95	0,97	1,00
	aerobic, room temperature	0,33	0,50	0,67	0,67	0,84	0,85	0,84	0,97	1,00	1,09	1,07	1,10	1,16	1,09	1,18	1,08	1,12	1,07	1,08
Bobek cones	anaerobic, cold room	0,16	0,18	0,19	0,21	0,22	0,21	0,24	0,24	0,25	0,25	0,24	0,24	0,23	0,22	0,26	0,26	0,30	0,30	0,24
	anaerobic, room temperature	0,16	0,19	0,19	0,23	0,24	0,25	0,25	0,26	0,32	0,35	0,37	0,30	0,30	0,36	0,35	0,36	0,44	0,52	0,51
	aerobic, cold room	0,16	0,22	0,18	0,24	0,28	0,30	0,28	0,31	0,29	0,30	0,30	0,34	0,33	0,32	0,36	0,41	0,39	0,37	0,32
	aerobic, room temperature	0,16	0,19	0,23	0,25	0,30	0,32	0,34	0,47	0,53	0,58	0,60	0,72	0,75	0,73	0,75	0,69	0,60	0,64	0,68
Bobek pellets	anaerobic, cold room	0,32	0,32	0,34	0,30	0,29	0,29	0,25	0,25	0,25	0,21	0,25	0,22	0,23	0,20	0,20	0,26	0,23	0,21	0,16
	anaerobic, room temperature	0,32	0,31	0,29	0,28	0,26	0,25	0,20	0,21	0,19	0,16	0,15	0,17	0,18	0,16	0,14	0,16	0,17	0,19	0,23
	aerobic, cold room	0,32	0,59	0,68	0,65	0,77	0,76	0,79	0,77	0,76	0,88	0,86	0,82	0,85	0,77	0,84	0,79	0,80	0,85	0,86
	aerobic, room temperature	0,32	0,62	0,69	0,74	0,85	0,82	0,81	0,84	0,87	0,81	0,70	0,76	0,78	0,81	0,69	0,68	0,62	0,55	0,57
Styrian Gold cones	anaerobic, cold room	0,43	0,42	0,43	0,48	0,45	0,48	0,50	0,53	0,52	0,57	0,62	0,67	0,72	0,56	0,62	0,61	0,68	0,61	0,50
	anaerobic, room temperature	0,43	0,46	0,48	0,50	0,53	0,55	0,53	0,55	0,58	0,60	0,80	0,79	0,85	0,79	0,71	0,66	0,75	0,83	0,88
	aerobic, cold room	0,43	0,50	0,51	0,51	0,53	0,49	0,57	0,51	0,59	0,57	0,68	0,63	0,76	0,67	0,76	0,70	0,71	0,87	0,74
	aerobic, room temperature	0,43	0,49	0,52	0,52	0,56	0,56	0,63	0,73	0,78	0,90	0,99	0,91	0,96	1,07	0,87	0,66	0,65	0,65	0,80
Savinjski golding pellets	anaerobic, cold room	0,12	0,12	0,13	0,14	0,14	0,14	0,14	0,10	0,08	0,07	0,06	0,06	0,05	0,07	0,08	0,07	0,09	0,10	0,07
	anaerobic, room temperature	0,12	0,16	0,14	0,11	0,13	0,14	0,14	0,15	0,20	0,21	0,21	0,22	0,20	0,20	0,21	0,20	0,24	0,24	0,24
	aerobic, cold room	0,12	0,12	0,15	0,19	0,19	0,21	0,21	0,20	0,20	0,21	0,22	0,23	0,23	0,23	0,23	0,24	0,24	0,22	0,21
	aerobic, room temperature	0,12	0,11	0,16	0,19	0,19	0,21	0,25	0,24	0,26	0,30	0,28	0,25	0,22	0,23	0,25	0,26	0,26	0,19	0,16
Styrian Wolf cones	anaerobic, cold room	0,43	0,46	0,54	0,57	0,54	0,52	0,54	0,53	0,52	0,52	0,52	0,66	0,56	0,57	0,66	0,64	0,72	0,79	0,79
	anaerobic, room temperature	0,43	0,45	0,55	0,57	0,53	0,56	0,56	0,57	0,61	0,68	0,85	0,78	0,67	0,72	0,75	0,73	0,90	0,98	0,98
	aerobic, cold room	0,43	0,48	0,62	0,62	0,61	0,66	0,64	0,67	0,71	0,75	0,75	0,84	0,72	0,70	0,80	0,86	0,92	0,95	0,97
	aerobic, room temperature	0,43	0,54	0,74	0,75	0,74	0,72	0,83	0,99	1,00	1,09	1,04	1,09	1,09	1,15	1,07	1,14	1,12	0,94	0,94
Strian Wolf pellets	anaerobic, cold room	0,63	0,56	0,56	0,56	0,56	0,57	0,60	0,56	0,62	0,55	0,62	0,56	0,59	0,56	0,61	0,62	0,62	0,58	0,55
	anaerobic, room temperature	0,63	0,54	0,53	0,53	0,55	0,55	0,54	0,53	0,47	0,58	0,57	0,53	0,56	0,50	0,55	0,60	0,64	0,63	0,64
	aerobic, cold room	0,63	0,80	1,07	1,31	1,55	1,56	1,57	1,63	1,72	1,74	1,77	1,80	1,77	1,76	1,48	1,51	1,50	1,43	1,55
	aerobic, room temperature	0,63	1,20	1,54	1,74	1,66	1,66	1,78	1,77	1,66	1,46	1,53	1,53	1,43	1,45	1,38	1,35	1,15	1,13	1,09

Supplementary Table S5. Changes in beta-caryophyllene content in all varieties for all storage conditions.

Variety/form	conditions \ time [months]	0	1	2	3	4	5	6	7	8	9	10	11	12	14	16	18	20	22	24
		Content of beta-caryophyllene [rel. %]																		
Celeia cones	anaerobic, cold room	8,50	8,41	8,01	8,25	8,18	8,13	8,51	8,15	8,58	8,38	8,13	8,25	7,87	8,23	8,51	8,78	8,28	8,43	8,40
	anaerobic, room temperature	8,50	8,37	8,39	8,39	8,27	8,39	8,46	8,41	8,74	8,48	8,89	8,98	8,73	8,94	8,77	9,32	8,61	8,48	8,55
	aerobic, cold room	8,50	8,74	8,76	8,46	8,52	8,09	8,37	8,02	8,06	8,28	7,95	7,55	7,58	7,62	7,04	7,37	6,57	6,25	5,85
	aerobic, room temperature	8,501	8,955	9,181	8,70	8,65	8,28	8,18	8,16	7,66	6,87	5,70	4,78	4,77	4,11	4,24	3,54	3,53	2,53	2,52
Celeia pellets	anaerobic, cold room	9,45	9,65	10,41	10,05	10,01	10,09	10,40	9,94	10,40	11,19	10,42	10,41	10,29	11,12	10,58	11,30	11,00	11,13	10,49
	anaerobic, room temperature	9,45	9,73	10,12	10,12	9,97	10,06	10,28	10,32	10,83	11,02	11,38	11,22	11,57	11,33	11,55	11,61	11,70	12,02	11,54
	aerobic, cold room	9,45	13,24	13,91	14,12	14,29	13,42	13,34	12,66	12,57	12,65	11,43	11,79	11,51	10,91	9,46	8,86	8,00	7,38	7,27
	aerobic, room temperature	9,45	13,29	14,08	14,63	13,90	13,49	12,39	12,80	11,72	10,90	8,69	7,24	6,99	6,08	5,52	5,28	3,25	2,01	1,66
Aurora cones	anaerobic, cold room	4,15	4,15	4,10	4,02	4,26	4,26	4,08	4,19	4,45	4,53	4,13	4,09	4,70	4,68	4,31	4,85	4,81	4,22	4,36
	anaerobic, room temperature	4,15	4,06	4,07	4,03	4,18	4,23	4,13	4,05	4,66	4,74	4,79	4,75	4,68	4,72	5,01	4,99	5,40	5,36	5,63
	aerobic, cold room	4,15	4,63	4,63	4,63	4,42	4,58	5,00	4,38	4,65	5,10	4,59	4,45	4,40	4,51	4,65	4,53	4,63	4,38	4,06
	aerobic, room temperature	4,15	4,67	4,58	4,37	4,40	4,45	4,61	4,74	4,76	4,04	3,55	3,67	3,43	2,83	2,51	2,17	1,58	1,21	1,12
Aurora pellets	anaerobic, cold room	4,26	4,75	4,71	4,59	4,84	4,93	4,80	4,99	5,06	5,10	5,31	5,04	5,41	5,48	5,95	5,34	5,74	5,45	5,46
	anaerobic, room temperature	4,26	4,84	4,73	4,61	4,68	4,70	4,99	5,13	5,21	5,38	5,78	5,47	5,31	5,59	5,64	5,91	6,11	6,07	6,34
	aerobic, cold room	4,26	5,93	7,85	8,59	8,54	9,12	9,14	9,08	9,34	9,64	9,42	9,16	9,48	9,44	8,87	8,57	8,37	7,74	7,69
	aerobic, room temperature	4,26	6,99	9,43	9,45	9,48	9,40	9,31	9,25	9,00	8,80	8,69	8,71	8,31	7,69	7,53	7,23	5,35	4,53	3,53
Bobek cones	anaerobic, cold room	3,66	3,80	4,02	3,69	3,84	3,91	3,97	4,04	4,99	5,35	3,85	3,83	3,92	3,75	4,25	4,01	4,50	4,39	4,18
	anaerobic, room temperature	3,66	3,85	3,99	3,86	3,87	3,89	3,68	4,32	4,50	4,86	4,25	3,73	3,94	3,67	3,95	4,48	4,71	5,11	4,80
	aerobic, cold room	3,66	4,47	4,23	3,97	4,44	4,17	4,05	4,40	5,15	4,81	4,01	3,57	3,54	3,52	3,23	3,90	3,60	3,72	3,46
	aerobic, room temperature	3,66	3,95	4,22	4,00	4,36	4,18	3,99	4,39	4,25	3,05	2,70	2,79	2,83	2,41	2,22	2,35	1,71	1,50	1,37
Bobek pellets	anaerobic, cold room	5,10	5,70	5,64	5,74	5,70	5,57	5,88	5,90	5,88	5,95	5,90	6,17	6,15	6,21	6,57	6,89	6,65	6,67	6,23
	anaerobic, room temperature	5,10	5,54	5,40	5,53	5,77	5,65	5,94	6,10	6,26	6,39	6,75	6,44	6,46	6,48	6,49	6,60	6,95	7,17	7,84
	aerobic, cold room	5,10	8,60	9,88	9,86	11,66	11,18	11,76	12,21	11,38	11,80	11,59	11,91	12,06	12,52	10,89	10,76	10,49	10,20	10,50
	aerobic, room temperature	5,10	9,60	10,25	11,13	12,25	11,63	11,20	9,52	6,10	3,34	2,04	1,86	1,67	1,47	0,96	0,57	0,50	0,47	0,56
Styrian Gold cones	anaerobic, cold room	9,79	10,28	10,21	9,38	9,92	9,88	10,34	10,34	10,89	10,59	11,05	10,71	12,31	12,16	12,07	11,29	10,96	10,68	10,58
	anaerobic, room temperature	9,79	10,76	10,16	9,79	9,97	9,82	10,55	10,51	10,77	10,30	9,90	10,34	11,13	9,34	10,44	10,17	10,54	10,11	8,53
	aerobic, cold room	9,79	11,01	10,81	9,76	10,15	9,89	10,83	10,67	11,26	10,78	10,23	10,04	11,61	10,09	10,08	10,41	10,08	9,93	9,23
	aerobic, room temperature	9,79	10,95	10,35	10,28	9,79	10,08	10,20	9,30	8,37	6,63	5,99	5,87	5,06	4,83	3,86	3,27	2,62	1,90	1,83
Savinjski golding pellets	anaerobic, cold room	11,34	11,08	11,40	11,00	11,87	11,32	11,17	11,70	11,68	11,70	12,41	12,59	12,25	12,29	12,61	12,71	12,90	13,10	12,13
	anaerobic, room temperature	11,34	11,36	11,47	11,22	11,34	11,55	11,59	11,93	12,16	12,22	12,70	12,34	12,31	12,77	12,85	13,12	13,40	12,81	13,08
	aerobic, cold room	11,34	11,13	14,81	15,55	15,48	15,11	15,31	15,06	15,26	15,35	14,92	14,97	14,65	15,17	15,29	13,82	13,87	13,79	13,10
	aerobic, room temperature	11,34	15,73	16,11	16,02	16,07	14,97	15,06	15,03	14,19	13,47	12,70	11,82	10,90	9,06	8,67	9,39	7,59	4,99	4,87
Styrian Wolf cones	anaerobic, cold room	3,58	3,44	3,79	3,81	3,78	3,62	3,72	3,82	3,77	3,68	3,73	3,80	3,92	4,02	4,33	4,42	4,59	4,64	4,06
	anaerobic, room temperature	3,58	3,32	3,77	3,65	3,64	3,69	3,69	3,81	4,01	3,97	4,10	4,11	4,12	4,22	4,40	4,53	5,08	4,89	4,74
	aerobic, cold room	3,58	3,70	3,90	3,85	3,84	3,93	3,90	3,91	3,97	3,95	4,03	3,98	3,60	3,53	3,39	3,24	3,18	2,75	2,82
	aerobic, room temperature	3,58	3,70	4,27	3,96	3,98	3,57	3,75	3,40	3,06	2,39	1,97	1,65	1,61	1,36	1,29	1,03	0,80	0,65	0,61
Strian Wolf pellets	anaerobic, cold room	3,77	3,53	3,63	3,53	3,57	3,57	3,81	3,72	4,06	3,86	4,02	3,62	4,03	4,13	4,41	4,33	4,06	3,98	3,98
	anaerobic, room temperature	3,77	3,35	3,52	3,49	3,51	3,63	3,83	3,76	4,14	4,06	3,99	3,82	4,16	4,17	4,34	4,45	4,70	4,75	4,67
	aerobic, cold room	3,77	4,58	6,08	6,67	7,38	7,09	7,38	7,36	7,22	7,03	6,90	6,68	5,81	5,36	4,79	4,20	3,23	3,16	2,77
	aerobic, room temperature	3,77	6,21	7,29	7,26	6,95	6,15	5,45	4,94	2,97	2,91	2,78	2,30	2,51	2,48	2,19	1,57	0,83	0,76	0,76

Supplementary Table S6. Changes in alpha-humulene content in all varieties for all storage conditions.

Variety/form	conditions \ time [months]	0	1	2	3	4	5	6	7	8	9	10	11	12	14	16	18	20	22	24
		Content of alpha-humulene [rel. %]																		
Celeia cones	anaerobic, cold room	16,30	16,33	15,63	16,11	15,99	16,21	17,07	16,26	17,34	17,04	16,58	17,30	16,18	17,70	18,00	18,55	17,56	17,91	18,14
	anaerobic, room temperature	16,30	16,43	16,46	16,85	16,58	17,29	17,43	17,48	18,14	18,03	19,22	19,94	20,05	20,16	19,46	21,05	20,54	20,30	20,88
	aerobic, cold room	16,30	16,63	16,97	16,64	17,08	16,18	16,92	16,09	16,59	17,06	16,47	18,00	19,19	16,00	15,26	16,41	14,49	14,20	13,37
	aerobic, room temperature	16,30	17,40	18,08	17,11	17,27	17,31	17,48	17,75	17,29	16,41	14,57	12,89	12,76	11,08	11,17	9,80	9,73	6,89	6,78
Celeia pellets	anaerobic, cold room	18,34	18,66	19,49	19,52	19,52	19,64	20,49	19,46	20,63	22,30	20,72	20,96	20,64	23,81	21,40	22,90	22,55	22,56	21,31
	anaerobic, room temperature	18,34	18,92	19,89	19,96	19,50	19,77	20,64	20,71	21,94	22,54	23,86	23,71	24,22	24,53	24,58	25,32	25,57	26,37	26,06
	aerobic, cold room	18,34	26,43	27,97	30,10	30,33	28,09	28,74	27,62	27,60	28,19	26,54	26,97	26,12	26,80	23,26	24,42	23,10	19,71	18,94
	aerobic, room temperature	18,34	26,48	28,69	30,70	29,04	29,35	28,53	28,68	28,06	26,24	23,07	20,28	19,76	17,49	16,75	16,35	10,59	6,92	5,29
Aurora cones	anaerobic, cold room	11,87	11,98	11,80	11,71	12,48	12,58	11,88	12,96	13,37	13,29	12,64	12,25	14,01	14,03	12,80	14,53	14,53	12,79	13,04
	anaerobic, room temperature	11,87	12,01	11,90	11,78	12,35	12,57	12,12	12,27	14,37	14,60	15,28	15,02	14,50	14,73	15,82	15,79	17,66	18,08	18,83
	aerobic, cold room	11,87	13,46	13,80	12,83	13,16	13,66	13,32	13,45	14,23	15,55	13,96	13,72	13,23	13,69	14,52	14,15	14,82	14,19	13,25
	aerobic, room temperature	11,87	13,80	14,05	13,18	14,18	13,75	14,30	15,27	16,11	14,85	13,65	13,60	12,86	10,89	9,70	8,35	6,37	4,81	4,22
Aurora pellets	anaerobic, cold room	12,53	14,04	13,88	13,60	14,37	15,00	14,25	14,93	15,25	15,36	16,20	15,24	16,35	16,44	18,10	16,16	17,60	16,66	16,71
	anaerobic, room temperature	12,53	14,35	14,00	13,70	13,95	14,35	15,06	15,80	16,22	17,14	18,71	17,41	16,93	17,78	18,04	19,14	20,43	20,81	21,64
	aerobic, cold room	12,53	17,65	23,28	26,31	26,61	28,88	28,74	29,14	29,56	31,68	31,54	30,54	32,31	31,95	31,04	31,76	30,53	29,27	28,94
	aerobic, room temperature	12,53	21,07	27,90	25,56	30,68	30,56	32,30	31,73	33,30	35,51	33,49	34,46	34,33	32,24	31,62	30,51	24,60	20,90	16,72
Bobek cones	anaerobic, cold room	9,16	9,60	10,33	9,36	9,83	10,17	10,30	10,51	10,42	10,32	10,27	10,10	10,43	9,89	11,43	10,74	12,29	12,11	11,19
	anaerobic, room temperature	9,16	9,75	10,22	9,84	9,94	10,20	9,57	11,52	12,26	13,79	12,49	10,58	11,04	10,51	11,42	12,89	14,18	15,96	14,76
	aerobic, cold room	9,16	11,35	10,89	10,31	11,63	11,22	10,77	11,85	13,29	13,28	11,27	9,96	9,97	9,98	9,44	11,35	10,75	11,33	10,57
	aerobic, room temperature	9,16	10,04	10,81	10,30	11,51	11,59	11,17	12,94	13,28	10,32	9,56	9,73	10,07	8,29	7,91	8,16	6,00	5,26	4,73
Bobek pellets	anaerobic, cold room	12,16	13,29	13,30	13,36	13,42	12,95	13,85	13,99	13,89	14,22	14,01	14,57	14,43	14,65	15,68	16,80	15,85	16,08	14,98
	anaerobic, room temperature	12,16	12,96	12,53	12,92	13,66	13,23	14,09	14,65	15,22	15,83	16,61	15,84	15,86	15,88	15,90	16,44	17,20	18,03	20,39
	aerobic, cold room	12,16	20,55	23,79	23,84	28,67	27,42	29,62	30,30	29,24	31,90	30,10	31,71	31,65	32,61	31,52	30,24	30,43	29,48	31,29
	aerobic, room temperature	12,16	22,15	24,78	27,56	31,81	29,89	30,22	26,95	18,76	11,70	7,34	5,72	5,30	5,00	3,24	1,76	1,34	1,57	1,71
Styrian Gold cones	anaerobic, cold room	19,10	20,33	20,16	18,59	19,78	19,53	21,08	20,90	22,29	22,34	23,26	22,17	25,73	25,87	25,34	23,94	23,23	22,58	21,98
	anaerobic, room temperature	19,10	21,59	20,29	19,49	20,26	19,68	21,78	21,65	23,32	23,18	22,69	22,63	25,39	21,10	23,09	24,18	24,75	24,59	24,23
	aerobic, cold room	19,10	22,02	21,68	19,53	20,73	19,77	22,09	21,76	23,44	23,17	21,91	21,31	25,00	22,03	22,09	23,38	22,32	22,36	20,49
	aerobic, room temperature	19,10	22,12	21,17	21,03	20,24	21,14	22,32	20,99	18,28	17,52	15,99	14,74	13,73	13,25	11,11	9,65	7,56	5,90	5,31
Savinjski golding pellets	anaerobic, cold room	30,62	30,19	30,77	30,33	32,37	31,20	30,31	32,16	32,24	32,50	35,17	35,38	34,46	34,59	35,41	35,84	37,99	37,99	34,40
	anaerobic, room temperature	30,62	30,20	31,36	28,44	29,66	31,83	31,92	33,58	34,71	36,13	37,97	37,98	36,48	37,40	38,55	39,19	42,02	40,86	40,64
	aerobic, cold room	30,62	30,24	40,73	37,79	44,65	45,67	44,38	44,02	45,03	45,78	44,92	45,76	44,71	45,84	46,62	44,19	44,59	44,31	43,25
	aerobic, room temperature	30,62	44,20	44,98	47,95	46,39	46,41	46,75	46,60	46,48	44,91	39,20	41,52	40,05	37,97	36,27	36,47	31,92	22,09	22,28
Styrian Wolf cones	anaerobic, cold room	8,24	8,14	8,89	8,87	8,81	8,56	8,68	9,01	8,98	8,69	9,00	10,57	9,28	9,50	10,46	10,65	11,22	11,57	10,18
	anaerobic, room temperature	8,24	7,85	8,90	8,65	8,60	8,82	8,77	9,15	9,86	9,87	10,95	11,57	10,17	10,42	11,19	11,45	13,56	13,79	13,02
	aerobic, cold room	8,24	8,22	9,32	9,15	9,23	10,00	9,44	9,64	9,79	9,75	10,38	10,13	9,06	8,93	8,69	8,65	8,68	7,82	7,75
	aerobic, room temperature	8,24	9,04	10,37	9,81	9,96	9,23	9,59	9,13	8,70	7,25	6,30	5,36	5,18	4,47	4,43	3,58	2,94	2,47	2,21
Strian Wolf pellets	anaerobic, cold room	9,43	8,74	8,96	8,78	9,07	8,92	9,57	9,33	10,31	9,82	10,22	9,00	10,12	10,34	11,20	11,19	10,30	10,14	9,99
	anaerobic, room temperature	9,43	8,36	8,86	8,84	9,04	9,26	9,79	9,70	10,71	10,78	10,59	9,96	10,88	10,91	11,40	11,94	12,90	13,15	12,79
	aerobic, cold room	9,43	11,47	15,46	17,48	20,16	19,16	19,97	20,30	20,36	19,97	19,75	19,64	17,38	16,74	14,74	13,51	9,38	10,47	10,66
	aerobic, room temperature	9,43	16,46	20,39	20,91	17,94	18,53	17,52	16,75	10,86	10,64	9,99	8,81	9,03	9,16	8,19	6,03	2,64	2,40	2,25

Supplementary Table S7. Changes in beta-farnesene content in all varieties for all storage conditions.

Variety/form	conditions \ time [months]	0	1	2	3	4	5	6	7	8	9	10	11	12	14	16	18	20	22	24
		Content of beta-farnesene [rel. %]																		
Celeia cones	anaerobic, cold room	4,72	4,51	4,47	4,29	4,22	4,22	4,33	4,22	4,35	4,32	4,20	3,97	3,90	3,93	3,95	4,07	3,74	3,96	3,80
	anaerobic, room temperature	4,72	4,27	4,41	4,24	4,21	4,21	4,10	3,81	4,13	3,62	3,45	3,24	3,38	3,14	3,09	3,13	2,26	1,98	2,01
	aerobic, cold room	4,72	4,58	4,51	4,27	4,30	3,97	4,04	3,76	3,88	3,86	3,67	3,27	3,08	3,11	2,74	2,68	2,18	2,17	2,10
	aerobic, room temperature	4,72	4,59	4,38	4,07	3,94	3,66	3,42	2,99	2,44	1,79	1,19	0,91	0,86	0,78	0,90	0,62	0,64	0,46	0,40
Celeia pellets	anaerobic, cold room	5,28	5,16	5,32	5,40	5,13	5,10	5,45	5,18	5,32	5,73	5,16	5,18	5,03	4,91	4,73	5,19	4,68	4,78	4,58
	anaerobic, room temperature	5,28	5,15	5,35	5,22	4,93	4,94	5,02	4,78	5,03	4,56	3,92	4,17	4,00	3,15	3,18	3,17	2,61	2,57	1,81
	aerobic, cold room	5,28	6,85	6,91	5,15	5,51	5,41	5,44	5,04	4,65	3,82	3,61	3,57	3,13	2,94	2,20	1,87	1,50	1,27	1,12
	aerobic, room temperature	5,28	6,61	6,58	5,74	5,95	4,87	3,98	3,23	2,65	1,60	1,02	0,76	0,65	0,61	0,58	0,54	0,38	0,26	0,25
Aurora cones	anaerobic, cold room	5,70	5,58	5,31	5,16	5,45	5,50	5,21	5,13	5,72	5,74	5,15	4,97	5,96	5,75	5,33	5,97	5,76	4,69	5,11
	anaerobic, room temperature	5,70	5,28	5,20	5,10	5,12	5,24	5,04	5,44	5,27	5,01	4,66	4,69	4,58	4,52	4,61	4,66	4,15	3,39	3,34
	aerobic, cold room	5,70	6,33	6,19	5,61	5,66	5,87	5,51	5,60	5,76	6,00	5,17	5,04	5,02	4,82	4,69	4,48	4,22	3,86	3,38
	aerobic, room temperature	5,70	6,12	5,93	5,52	5,47	4,90	4,95	4,50	3,59	2,23	1,41	1,26	1,27	0,87	0,71	0,53	0,36	0,45	0,40
Aurora pellets	anaerobic, cold room	5,79	6,34	6,19	5,87	6,20	6,48	6,12	6,42	6,41	6,39	6,56	6,33	6,58	6,55	7,11	6,31	6,51	6,33	6,22
	anaerobic, room temperature	5,79	6,38	6,14	5,92	5,90	5,98	6,13	6,19	6,09	6,02	6,09	5,80	5,58	5,59	5,41	5,76	5,34	5,05	4,85
	aerobic, cold room	5,79	7,72	9,60	10,11	9,61	9,58	9,40	8,80	8,72	7,91	6,95	6,39	5,61	5,35	4,00	2,83	2,32	1,72	1,35
	aerobic, room temperature	5,79	8,79	10,46	9,66	9,93	8,30	7,93	6,81	4,89	3,31	2,67	2,23	1,89	1,35	1,10	0,88	0,47	0,50	0,42
Bobek cones	anaerobic, cold room	4,52	4,53	4,59	4,23	4,29	4,52	4,34	4,43	4,43	4,00	3,94	4,10	4,21	3,95	4,34	3,77	4,23	3,66	4,02
	anaerobic, room temperature	4,52	4,63	4,44	4,27	4,22	4,30	4,01	4,50	4,50	4,64	3,10	3,05	3,23	2,66	2,84	3,41	2,72	2,50	2,36
	aerobic, cold room	4,52	5,05	4,78	4,49	4,50	4,58	4,20	4,25	4,50	4,69	3,50	3,06	2,98	2,75	2,38	2,68	2,31	1,88	1,97
	aerobic, room temperature	4,52	4,73	4,87	4,54	4,39	4,21	3,46	3,03	2,19	1,18	0,87	0,81	0,84	0,18	0,21	0,25	0,24	0,26	0,22
Bobek pellets	anaerobic, cold room	5,14	5,58	5,19	5,47	5,50	5,34	5,65	5,60	5,59	5,55	5,51	5,81	5,74	5,72	5,98	5,64	6,05	5,95	5,52
	anaerobic, room temperature	5,14	5,39	5,11	5,21	5,50	5,27	5,47	5,46	5,38	5,17	5,38	5,14	4,96	5,00	5,12	4,90	5,17	4,82	4,15
	aerobic, cold room	5,14	7,96	8,15	8,21	9,42	8,92	8,65	8,76	8,06	8,46	7,42	6,63	7,31	6,64	5,83	5,19	4,50	4,12	2,32
	aerobic, room temperature	5,14	8,15	8,50	8,42	8,37	6,92	4,80	2,06	0,31	0,35	0,34	0,32	0,33	0,33	0,34	0,31	0,36	0,34	0,35
Styrian Gold cones	anaerobic, cold room	5,13	4,87	4,86	4,49	4,88	4,64	4,76	4,73	4,87	4,30	4,06	4,54	4,42	4,72	4,64	4,10	3,97	3,75	4,24
	anaerobic, room temperature	5,13	4,99	4,86	4,62	4,73	4,28	4,32	4,27	3,88	3,26	2,40	2,41	2,43	2,53	2,50	2,49	2,39	2,06	1,83
	aerobic, cold room	5,13	5,16	5,06	4,67	4,86	4,63	4,98	4,95	4,86	4,23	3,76	3,41	3,16	3,10	3,06	2,90	2,94	2,60	2,46
	aerobic, room temperature	5,13	4,98	4,95	4,80	4,54	4,26	3,74	3,02	2,20	1,31	1,02	1,16	0,65	0,74	0,61	0,52	0,42	0,11	0,18
Savinjski golding pellets	anaerobic, cold room	6,47	6,26	6,46	5,65	5,82	6,12	6,09	6,21	6,19	6,17	6,05	6,51	6,35	6,10	6,16	6,06	5,90	5,31	5,47
	anaerobic, room temperature	6,47	6,40	6,37	5,97	6,00	6,02	5,94	5,80	5,47	4,68	4,51	4,45	4,43	4,20	3,84	3,88	3,00	2,32	2,53
	aerobic, cold room	6,47	6,38	8,19	8,01	7,82	7,80	7,64	7,65	7,91	6,86	6,58	6,50	6,48	6,50	5,18	5,44	5,20	4,32	4,05
	aerobic, room temperature	6,47	8,61	8,73	8,60	8,42	7,05	6,54	6,13	5,35	3,96	2,48	2,55	2,47	2,26	1,84	1,43	0,25	0,25	0,26
Styrian Wolf cones	anaerobic, cold room	5,70	5,44	5,55	5,47	5,57	5,46	5,52	5,66	5,43	5,43	5,23	5,97	5,64	5,39	5,86	5,96	5,33	5,66	5,00
	anaerobic, room temperature	5,70	5,20	5,49	5,23	5,04	5,22	5,07	5,00	4,82	4,37	3,59	4,20	4,17	3,86	3,94	4,14	3,43	2,98	2,85
	aerobic, cold room	5,70	5,37	5,39	5,18	5,13	4,98	4,82	4,72	4,56	4,50	3,95	3,42	3,91	3,52	2,99	2,87	2,34	1,93	1,99
	aerobic, room temperature	5,70	5,56	5,20	4,77	4,43	4,14	3,51	2,74	2,07	1,24	0,81	0,39	0,75	0,36	0,41	0,40	0,36	0,59	0,31
Strian Wolf pellets	anaerobic, cold room	6,56	6,07	6,05	6,01	6,08	6,10	6,22	6,21	6,89	6,29	6,63	5,84	6,18	6,44	6,61	6,62	5,96	5,81	5,75
	anaerobic, room temperature	6,56	5,69	5,80	5,82	5,87	5,92	6,05	5,84	5,93	5,84	5,69	5,27	5,43	5,59	5,63	5,64	5,22	4,99	4,78
	aerobic, cold room	6,56	7,32	8,36	8,50	4,98	4,77	5,16	4,33	3,72	2,59	2,26	1,64	1,10	0,89	0,45	0,45	0,24	0,33	0,41
	aerobic, room temperature	6,56	7,53	5,34	4,23	1,96	1,52	0,77	0,40	0,31	0,30	0,42	0,60	0,54	0,54	0,62	0,61	0,68	0,68	0,68

Supplementary table S8. Changes in caryophyllene-oxide content in all varieties for all storage conditions.

Variety/form	conditions \ time [months]	0	1	2	3	4	5	6	7	8	9	10	11	12	14	16	18	20	22	24
		Content of caryophyllene-oxide [rel. %]																		
Celeia cones	anaerobic, cold room	0,55	0,78	0,67	0,91	1,01	0,95	1,35	1,04	1,20	1,26	1,22	1,52	1,36	1,60	1,64	1,61	1,75	1,59	1,59
	anaerobic, room temperature	0,55	0,91	0,76	0,97	1,16	1,22	1,53	1,41	1,34	1,62	1,52	1,77	2,24	1,33	1,77	1,86	1,93	1,75	2,02
	aerobic, cold room	0,54	0,83	0,93	1,10	1,25	1,20	1,59	1,48	1,68	1,96	1,99	2,15	1,72	2,27	2,45	2,74	2,92	3,03	3,40
	aerobic, room temperature	0,55	0,93	1,32	1,68	1,76	1,84	2,51	2,60	2,97	2,99	2,50	2,45	2,13	2,55	2,44	2,40	2,11	1,98	1,82
Celeia pellets	anaerobic, cold room	0,92	0,98	1,28	1,05	1,12	1,12	1,19	0,98	1,24	1,16	1,11	1,15	1,23	1,19	1,26	1,34	1,54	1,45	1,45
	anaerobic, room temperature	0,92	1,01	1,24	1,20	1,10	1,14	1,48	1,30	1,24	1,15	1,61	1,20	1,33	1,84	1,82	1,95	2,31	1,86	2,54
	aerobic, cold room	0,92	1,67	2,09	2,06	2,52	2,78	2,86	2,89	3,04	3,52	3,62	3,63	3,98	4,51	5,18	5,04	5,40	5,85	6,05
	aerobic, room temperature	0,92	1,77	2,60	2,21	2,39	3,05	3,40	3,37	3,78	4,32	4,83	5,58	5,76	6,03	6,45	6,32	7,28	7,34	7,33
Aurora cones	anaerobic, cold room	0,32	0,31	0,32	0,32	0,35	0,32	0,34	0,35	0,28	0,16	0,32	0,28	0,15	0,17	0,20	0,24	0,26	0,24	0,22
	anaerobic, room temperature	0,32	0,30	0,33	0,33	0,33	0,38	0,38	0,31	0,38	0,25	0,42	0,40	0,19	0,28	0,33	0,33	0,45	0,52	0,50
	aerobic, cold room	0,32	0,37	0,40	0,41	0,45	0,43	0,41	0,43	0,31	0,26	0,21	0,42	0,21	0,30	0,32	0,38	0,56	0,59	0,52
	aerobic, room temperature	0,32	0,39	0,43	0,49	0,55	0,54	0,60	0,53	0,81	1,11	1,10	0,87	0,66	0,88	1,09	1,14	1,33	1,39	1,28
Aurora pellets	anaerobic, cold room	0,39	0,38	0,38	0,39	0,40	0,37	0,34	0,12	0,16	0,16	0,19	0,18	0,13	0,11	0,19	0,26	0,34	0,26	0,28
	anaerobic, room temperature	0,39	0,36	0,38	0,35	0,34	0,36	0,38	0,28	0,12	0,25	0,19	0,11	0,10	0,12	0,33	0,33	0,41	0,42	0,43
	aerobic, cold room	0,39	0,52	0,82	1,01	0,87	0,85	0,84	0,46	0,39	0,68	1,04	0,81	0,56	0,52	0,70	0,90	0,90	1,01	0,96
	aerobic, room temperature	0,39	0,63	0,80	0,96	1,00	1,06	1,08	0,82	0,70	0,58	0,58	0,51	0,45	0,57	0,61	0,62	0,88	1,05	1,04
Bobek cones	anaerobic, cold room	0,32	0,43	0,59	0,54	0,60	0,68	0,71	0,64	0,77	0,74	0,67	0,60	0,62	0,58	0,18	0,18	0,23	0,25	0,73
	anaerobic, room temperature	0,32	0,42	0,59	0,63	0,62	0,71	0,63	0,77	1,24	1,50	1,37	0,91	0,89	1,01	1,11	1,15	1,46	1,40	1,39
	aerobic, cold room	0,32	0,71	0,73	0,69	0,97	1,09	0,92	1,32	0,72	1,20	1,14	1,06	1,20	1,37	1,46	1,66	1,98	2,43	2,12
	aerobic, room temperature	0,32	0,50	0,65	0,62	0,95	1,22	1,37	2,08	2,19	2,96	2,22	1,70	1,77	1,86	1,78	1,70	1,59	1,37	1,41
Bobek pellets	anaerobic, cold room	0,92	0,46	0,53	0,38	0,45	0,34	0,31	0,35	0,33	0,33	0,40	0,31	0,32	0,33	0,33	0,70	0,38	0,42	0,31
	anaerobic, room temperature	0,92	0,41	0,34	0,35	0,45	0,33	0,24	0,30	0,29	0,35	0,37	0,31	0,40	0,33	0,33	0,38	0,35	0,40	0,69
	aerobic, cold room	0,92	0,93	1,18	1,18	1,58	1,40	1,75	1,85	2,45	1,90	2,16	2,10	2,21	1,96	2,77	2,69	3,07	2,77	3,35
	aerobic, room temperature	0,92	1,00	1,19	1,67	1,89	2,26	2,94	3,82	5,12	6,24	7,69	7,93	7,62	8,27	8,48	8,65	7,06	6,88	7,06
Styrian Gold cones	anaerobic, cold room	0,30	0,54	0,49	0,46	0,53	0,47	0,61	0,54	0,72	0,84	1,11	0,77	1,33	0,96	1,02	1,03	1,15	1,16	0,88
	anaerobic, room temperature	0,30	0,53	0,54	0,49	0,61	0,57	0,94	0,74	1,03	1,67	1,86	2,00	2,20	1,42	1,26	1,24	1,53	1,62	1,38
	aerobic, cold room	0,30	0,56	0,61	0,55	0,63	0,51	0,73	0,61	0,92	1,11	1,19	1,01	1,94	1,33	1,82	2,36	2,13	2,36	1,65
	aerobic, room temperature	0,30	0,68	0,64	0,64	0,83	0,94	1,43	1,48	1,96	2,09	2,16	2,33	2,47	2,11	2,11	1,52	1,27	1,15	1,31
Savinjski golding pellets	anaerobic, cold room	0,38	0,60	0,53	0,49	0,51	0,58	0,42	0,46	0,38	0,52	0,68	0,66	0,52	0,59	0,64	0,60	0,88	0,79	0,68
	anaerobic, room temperature	0,38	1,00	0,51	0,55	0,60	0,65	0,49	0,50	0,55	0,82	0,88	0,98	0,85	0,94	1,10	1,15	1,13	1,54	1,39
	aerobic, cold room	0,38	0,61	0,76	0,81	0,87	1,03	0,88	1,07	0,98	1,09	1,39	1,32	1,06	1,16	1,34	1,38	1,67	1,81	1,91
	aerobic, room temperature	0,38	1,11	0,88	0,96	0,87	1,09	1,05	1,25	1,12	1,41	2,05	1,90	2,17	2,19	2,45	2,50	3,25	4,20	4,27
Styrian Wolf cones	anaerobic, cold room	0,28	0,44	0,36	0,36	0,29	0,36	0,30	0,18	0,19	0,18	0,20	0,29	0,18	0,20	0,24	0,24	0,27	0,31	0,35
	anaerobic, room temperature	0,28	0,68	0,34	0,36	0,33	0,43	0,34	0,19	0,20	0,27	0,47	0,35	0,22	0,23	0,32	0,32	0,43	0,55	0,50
	aerobic, cold room	0,28	0,56	0,47	0,49	0,49	0,54	0,58	0,42	0,44	0,31	0,85	0,83	0,47	0,47	0,48	0,56	0,77	1,02	0,69
	aerobic, room temperature	0,28	0,65	0,65	0,76	0,75	0,80	0,83	0,60	0,63	0,80	0,94	0,97	0,90	0,94	1,01	0,98	1,10	1,11	1,07
Strian Wolf pellets	anaerobic, cold room	0,35	0,30	0,29	0,31	0,40	0,32	0,11	0,19	0,23	0,20	0,21	0,17	0,13	0,13	0,26	0,24	0,24	0,19	0,20
	anaerobic, room temperature	0,35	0,28	0,30	0,30	0,29	0,30	0,20	0,20	0,19	0,24	0,23	0,21	0,24	0,22	0,24	0,26	0,33	0,29	0,30
	aerobic, cold room	0,35	0,47	0,59	0,97	1,50	1,29	0,81	0,78	1,03	1,23	1,44	1,41	1,54	1,78	1,94	2,22	2,30	2,47	2,53
	aerobic, room temperature	0,35	0,98	1,28	1,52	2,18	1,94	1,13	1,07	2,06	1,90	1,47	1,31	1,96	1,89	1,41	1,45	1,84	1,75	1,84

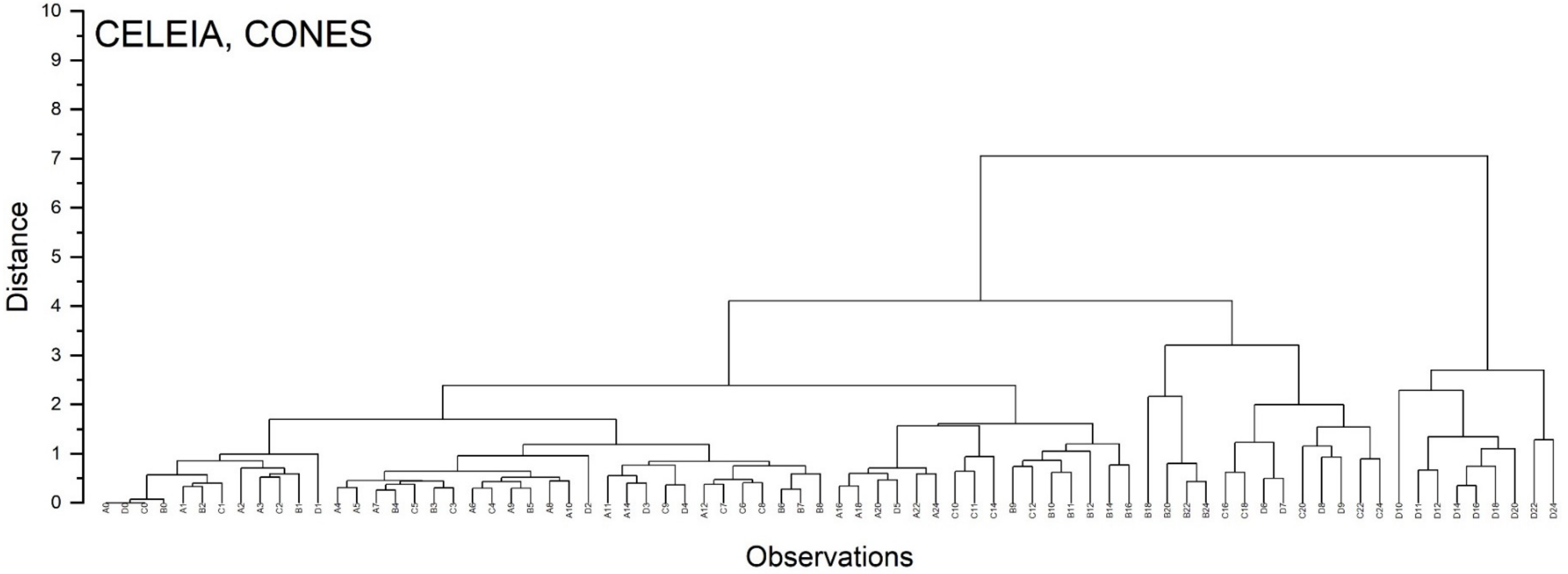

Supplementary figure S1: Dendrogram for cones of Celeia.

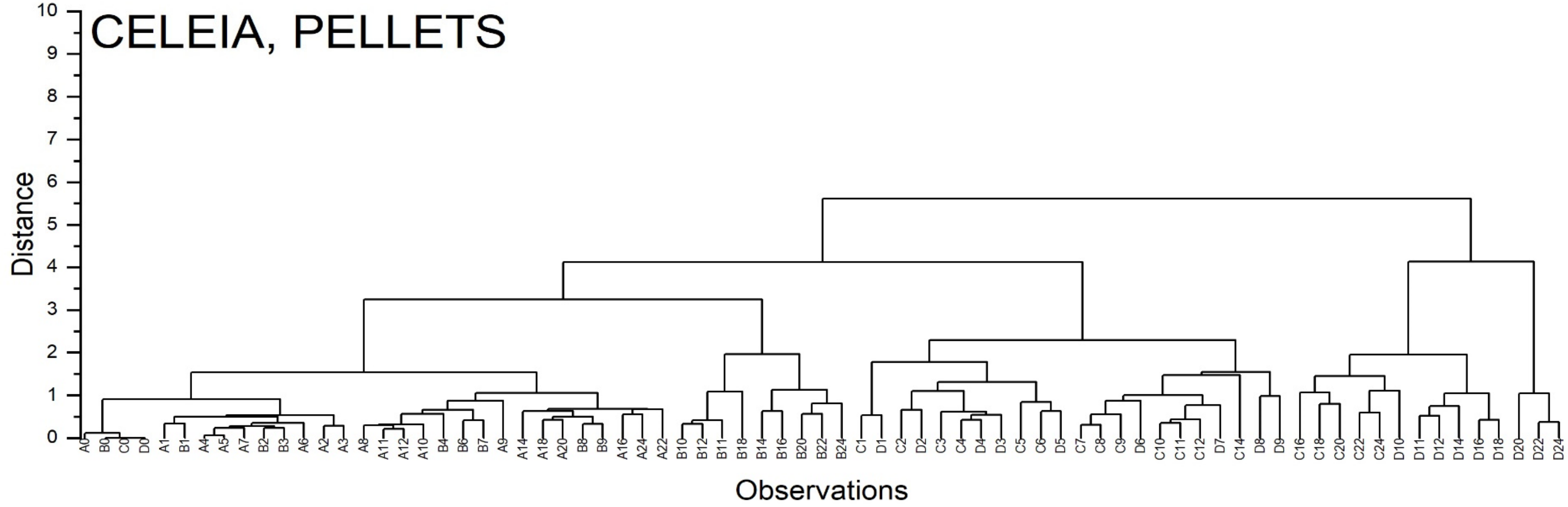

Supplementary figure S2: Dendrogram for pellets of Celeia

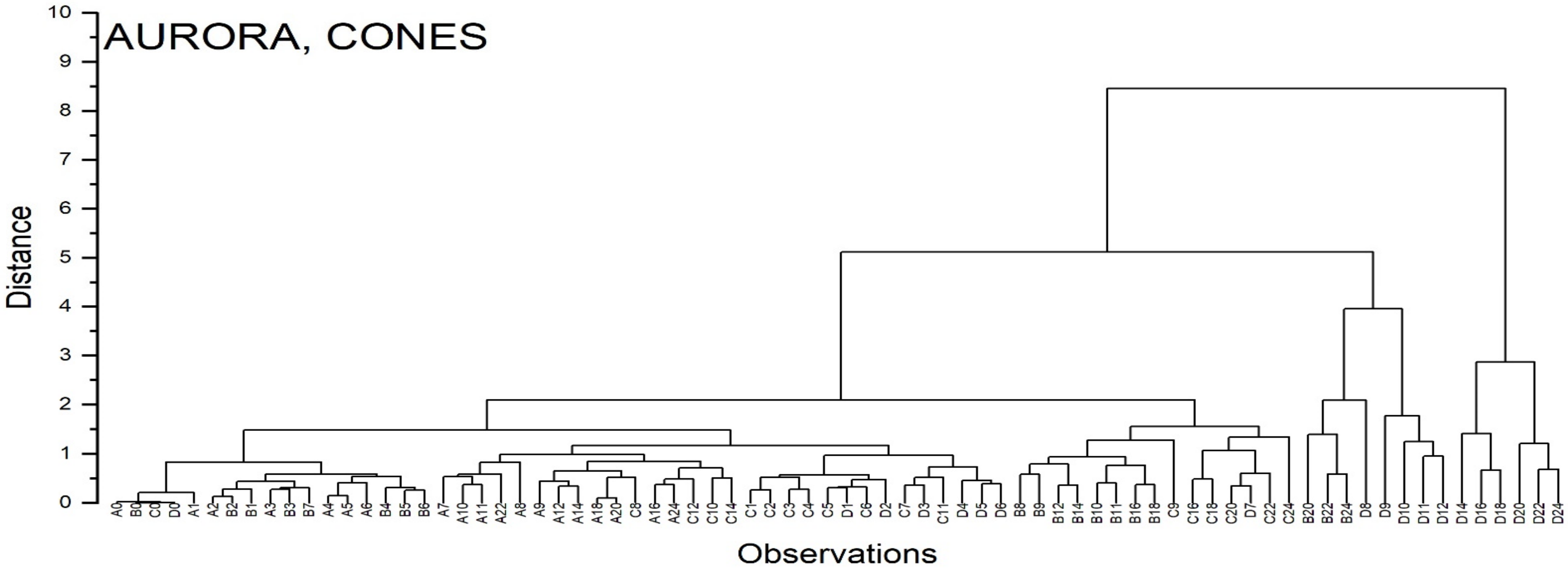

Supplementary figure S3: Dendrogram for cones of Aurora.

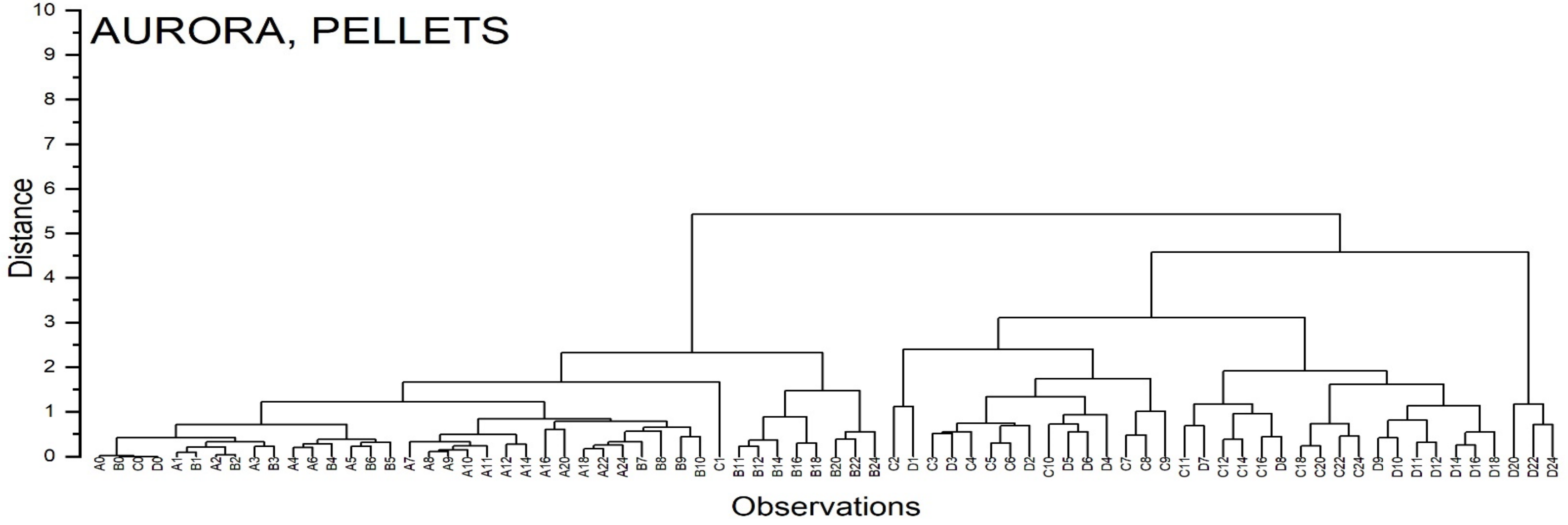

Supplementary figure S4: Dendrogram for pellets of Aurora.

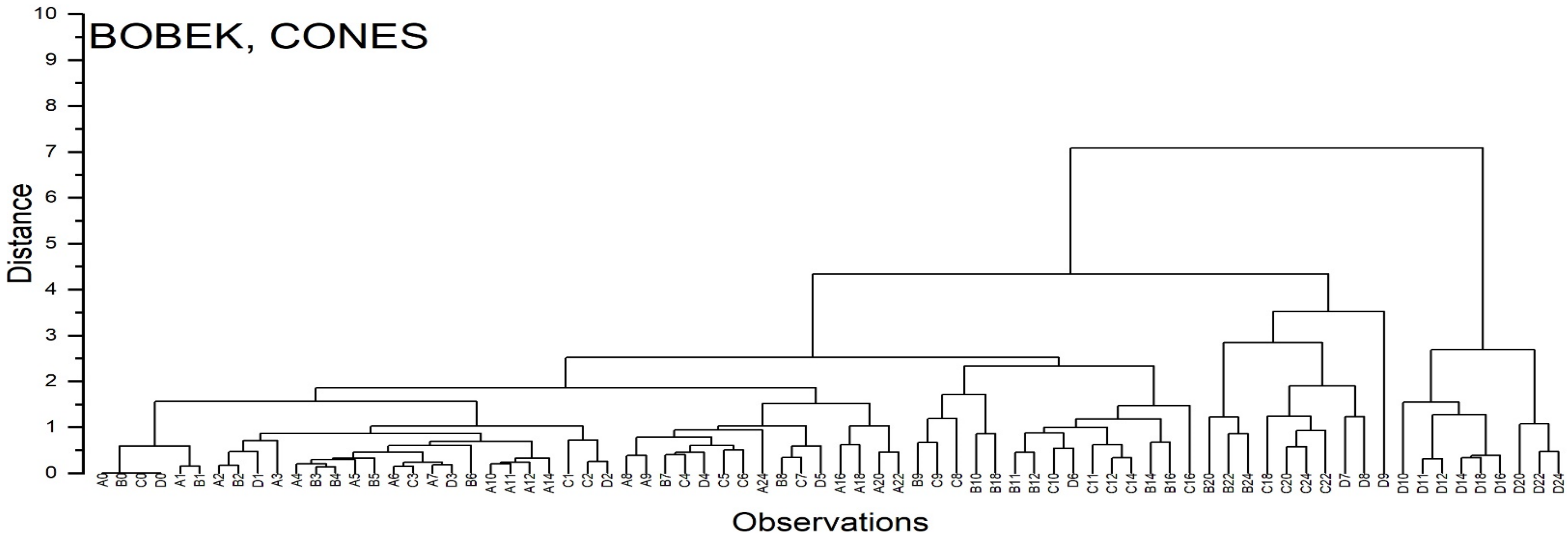

Supplementary figure S5: Dendrogram for cones of Bobek.

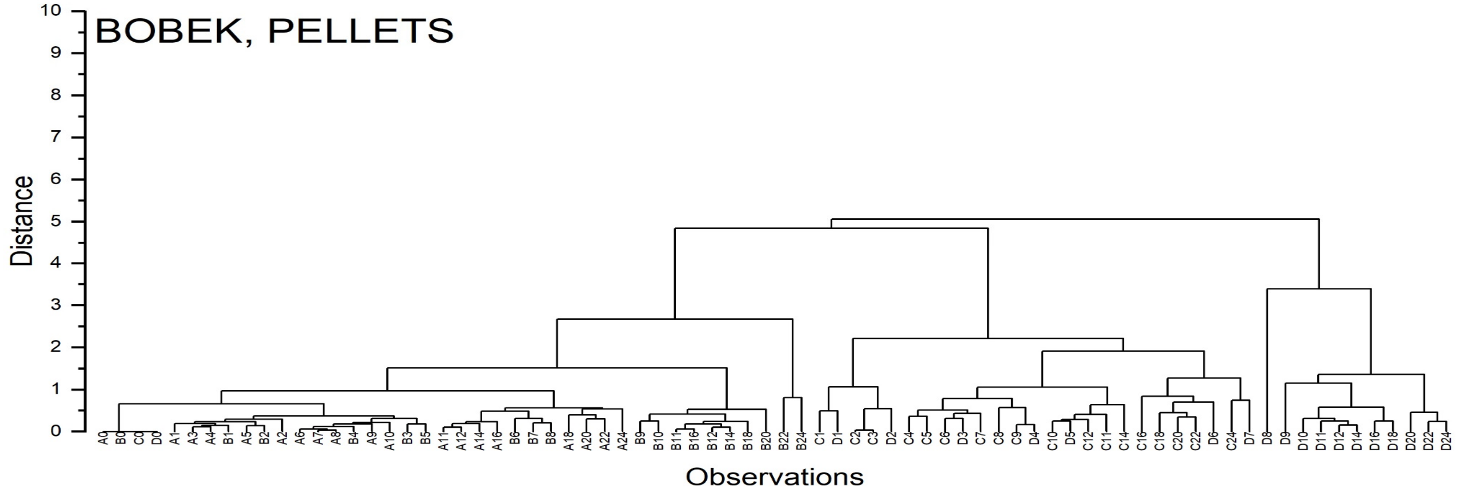

Supplementary figure S6: Dendrogram for pellets of Bobek.

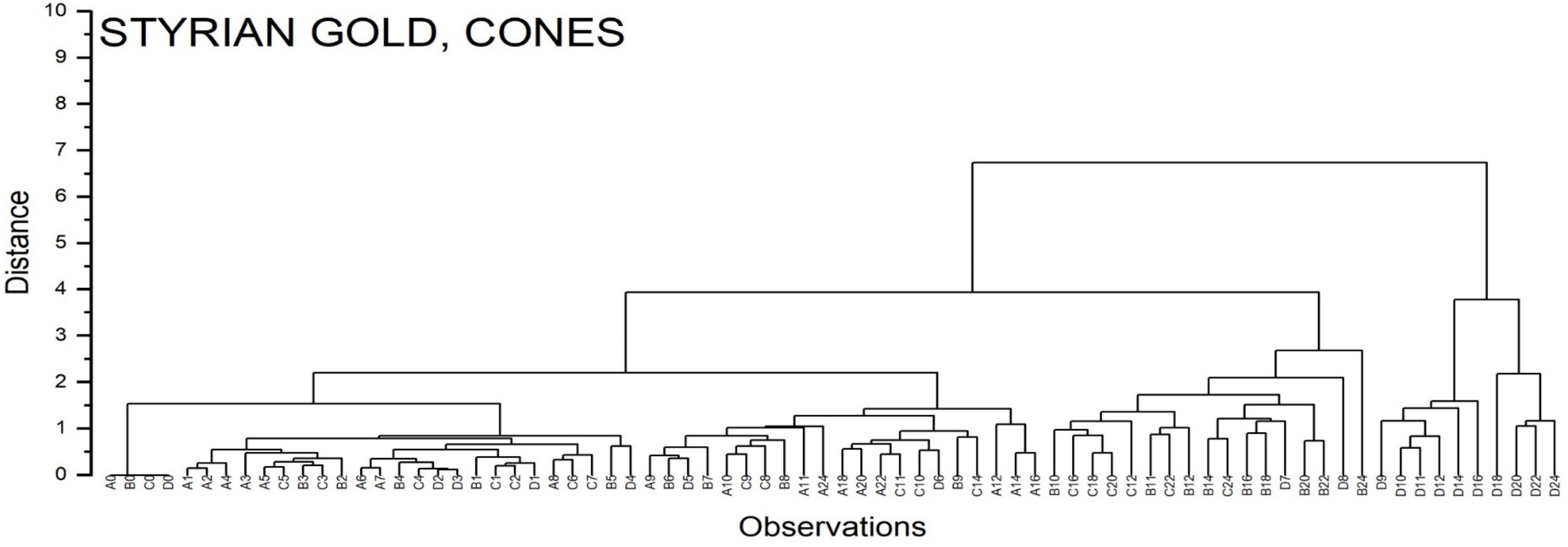

Supplementary figure S7: Dendrogram for cones of Styrian Gold

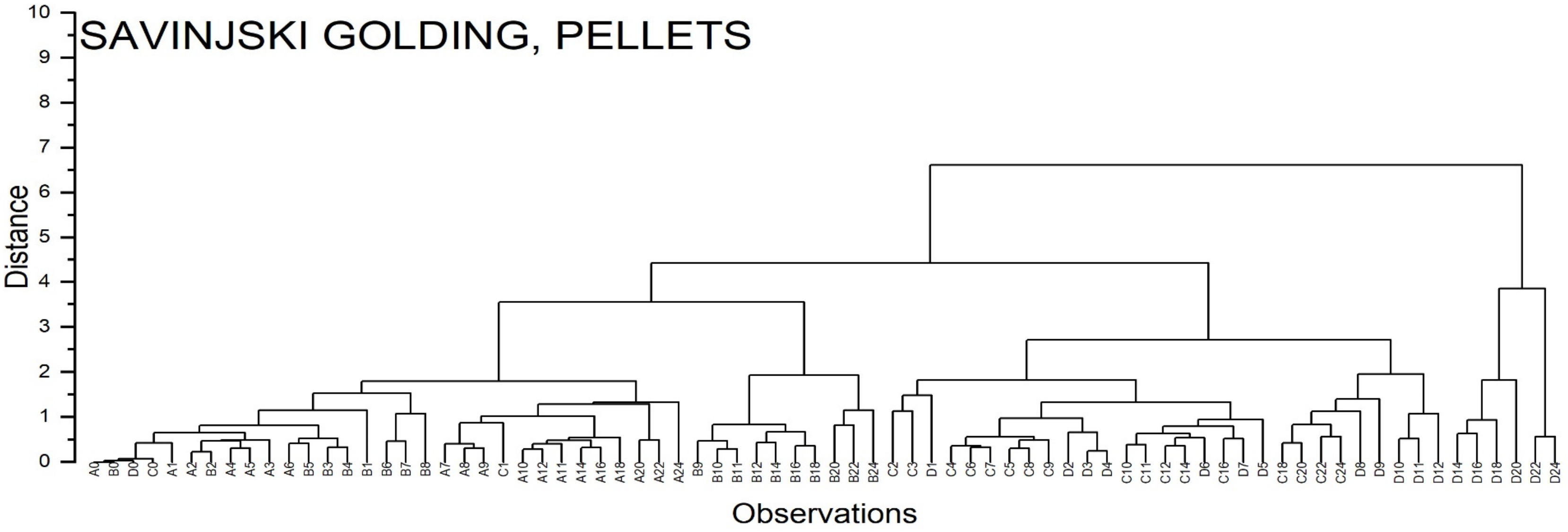

Supplementary figure S8: Dendrogram for pellets of Savinjski Golding.
